# How participants engage with emotion-focused training for couple identity (EFT-CIDE), a 14-day self-guided mobile app intervention: a framework analysis

**DOI:** 10.3389/fpsyg.2026.1877423

**Published:** 2026-07-15

**Authors:** Júlia Halamová

**Affiliations:** Institute of Applied Psychology, Faculty of Social and Economic Sciences, Comenius University in Bratislava, Bratislava, Slovakia

**Keywords:** couple intervention, emotion-focused therapy for couples, framework analysis, identity-system work, longitudinal qualitative research, respect, self-guided mobile-app intervention

## Abstract

**Background:**

In emotion-focused therapy for couples (EFT-C), identity is one of three relational systems alongside attachment and attraction. Emotion-Focused Training for Couple Identity (EFT-CIDE) is the first self-guided mobile app intervention targeting the identity system. Its quantitative outcomes are reported in a parallel manuscript. Although qualitative engagement work exists for other digital couple interventions, none has examined participant engagement with identity system intervention content in a self-guided mobile app format. In addition, qualitative engagement studies of digital couple interventions typically rely on post-hoc interviews; analysis of reflective text participants produce within the app itself remains uncommon.

**Methods:**

We analysed reflective text written by 60 Slovak participants during the 14-day EFT-CIDE intervention (2,740 indexed segments). Coding combined deductive anchors from EFT-C and the respect literature with inductive codes admitted via a watchlist, using the Framework Method with a longitudinal layer. Cross-case patterns were paired with within-case trajectories across the 14-day window.

**Results:**

Five backbone codes had high reach across the dataset: active listening (90%), giving appreciation (88%), constructive expression (85%), repair as growth (75%), and healthy boundary setting (67%). Three asymmetric splits characterised most participants: healthy boundary setting dominated over both internal and external blocks (13:1:1), mutual cycle attribution dominated over self-blame (9:1), and repair as growth dominated over over-responsibility (8:1). Six longitudinal trajectory types organised the dataset; 32 of 60 participants reached an integrative day-14 closure (“change starts with me”).

**Conclusion:**

EFT-CIDE appears to work well for participants showing open engagement or distress followed by recovery, and may serve as a useful adjunct for those in sustained distress. A smaller group of participants did not engage substantively with the app format.

## 1 Introduction

### 1.1 Identity in close relationships

Identity and respect are parallel terms for the same relational construct. One is rooted in emotion-focused therapy for couples (EFT-C), the other in close relationships research, and both designate a construct distinct from attachment (security and connection) and attraction (liking and desire). Within EFT-C, (Greenberg and Goldman 2008; Goldman and Greenberg, 2013) treat identity as one of three coordinate motivational systems alongside attachment and attraction. The close relationships respect literature articulates the parallel construct: Frei and Shaver (2002) identified it as multidimensional and organised around caring about the partner, valuing partner qualities, listening attentively, accepting differences, and refraining from contempt. Hendrick and Hendrick (2006) developed a self-report measure of respect in close relationships and demonstrated its discriminant validity against neighbouring constructs, with caring and supportiveness as central components. Hendrick et al. (2010) extended this work to family relationships, articulating respect as a relational practice that operates within identity, attachment, and intergenerational dimensions of family life. Lawrence-Lightfoot's (2000) philosophical exploration of respect provided a complementary articulation, treating respect as a relational practice cultivated through specific dispositions (empathy, dialogue, attention, healing), rather than as a static attitude or character trait.

Identity/respect operates across three levels. Cognitively, it includes recognising partner perspectives as legitimate even when not agreed with, acknowledging partner competence, attributing good intent, and recognising partner autonomy and self-determination. Affectively, it includes valuing partner qualities, admiration for the partner as a distinct person, and the absence of contempt. Behaviourally, it includes attentive listening, validation, appreciation, autonomy support, and articulation of one’s own legitimate limits. Its distinctness from attachment and attraction is empirically established, and it is theorised as a precondition for the relational work that produces attachment security and dyadic functioning over time (Frei and Shaver, 2002; Hendrick et al., 2010). Intervention content specifically targeting identity/respect remains comparatively limited. In most existing interventions, identity/respect features as one component among many, or attention is directed solely at attachment. Qualitative analysis of how identity/respect surfaces in everyday couple discourse during structured intervention is rare.

### 1.2 Emotion-focused therapy for couples and the identity system

Emotion-focused therapy for couples (EFT-C; Greenberg and Goldman, 2008) provides a theoretical framework within which respect can be located as an identity system construct. The framework treats couple difficulties as arising from disturbances across three coordinate motivational systems: identity (concerned with worth, acceptance, and status), attachment (concerned with felt safety, accessibility, and proximity), and attraction or liking (concerned with affection, desire, and pleasurable engagement). The three systems operate in parallel, not hierarchically. Intervention proceeds by identifying which system is the source of distress for a given couple at a given moment, instead of treating any one system as the default entry point.

In the multi-motivational framework (Goldman and Greenberg, 2013), identity system processes are treated as coordinate with attachment and attraction processes, supporting and supported by attachment system work, rather than subordinate to it. This positioning has both theoretical and clinical consequences. Theoretically, it allows respect-focused intervention to be conceptualised as a substantive intervention modality in its own right, not merely as an indirect route to attachment. Clinically, it suggests that identity system difficulties (corrosion of self-respect through chronic disrespect, erosion of partner respect through accumulated grievance, asymmetric blocked or refused boundary setting, sustained trust deficit, and over-responsibility) may warrant intervention components specifically designed for those difficulties, instead of being addressed solely through attachment system work.

### 1.3 The EFT-CIDE intervention

Mobile app and web-based delivery of couple interventions has broadened access to relational support beyond face-to-face therapy, enabling self-guided engagement at scale and at lower cost (Doss et al., 2013, 2017; Hatch et al., 2022; Roddy et al., 2020). The EFT-CIDE intervention (Halamová, 2026) is a 14-day self-guided mobile app intervention developed within the EFT-C framework specifically to deliver respect/identity system content. The intervention’s design parallels two companion interventions: EFT-CATT, an attachment system intervention focused on closeness (Halamová and Kernová, 2026), and EFT-CATR, an attraction system intervention focused on liking (Halamová and Hájková, 2026). Together, the three interventions instantiate the multi-motivational structure of EFT-C theory at the level of self-guided digital intervention, allowing empirical examination of whether identity system, attachment system, and attraction system processes can be addressed through structurally parallel reflective formats. The intervention was also tested in a separate randomised trial with a waitlist control group (Halamová, 2026). In that trial, participants reported feeling more respected by their partner immediately after the programme (BLRI Regard, d ≈ 0.31). At 1-month follow-up, the gains were larger for overall relationship adjustment (RDAS total, d ≈ 0.44) and for congruence. Effects were strongest for the measures closest to the identity system, smaller for the attachment measure (BARE), and absent for sexual satisfaction. The trial shows that the programme changes these scores; it cannot show how participants engaged with the content, or why it helped some people and not others. The present study examines this. Section 3.2 shows which content most participants took up, and Sections 3.4 and 3.6 show for whom the content became usable and for whom it did not. Together, these help explain why the trial’s effects varied between participants and why they were stronger for identity-related measures. We use the term *intervention* as is standard in this field, but EFT-CIDE is a short, self-guided reflection programme rather than therapy delivered by a clinician; the conclusions about whom it suits (see sections 4.4, 4.7) should be read with that in mind.

### 1.4 Research gap: qualitative analysis of intervention engagement

Quantitative outcome studies of self-guided couple interventions can establish whether and to what degree pre–post change occurs across measured outcomes. However, they cannot directly address the engagement processes that produce, or fail to produce, that change (Roddy et al., 2020; Linardon and Fuller-Tyszkiewicz, 2020). Three engagement phenomena are qualitatively distinct from outcome scores: within-individual response over time, across-participant variability in how the same intervention component is received, and the boundary conditions for intervention suitability. Outcome scores, which offer only a snapshot, do not capture these in the detail required to inform intervention design (Linardon and Fuller-Tyszkiewicz, 2020; Nwosu et al., 2022). Qualitative engagement work on digital couple interventions has typically used post-hoc semi-structured interviews (e.g., Aicken et al., 2025; Roddy et al., 2020). Analysis of the reflective text participants produce within the app itself remains uncommon, particularly for content targeting the identity system. To verify this, we scanned the literature on digital and app-based couple interventions for qualitative work on how participants engage with these programmes. Most evaluations are quantitative randomized trials, and the qualitative work relies on post-programme interviews, app usage data, or participants’ feedback about the programme, rather than on the reflective text they produce inside it (e.g., Aicken et al., 2025; Roddy et al., 2020). We did not find a study that analyses participants’ own in-app written reflections as data on how they engage with an intervention’s content, and none that does so for identity system content. We therefore present this as a gap that the present study addresses, rather than as proof that no such work exists. Two methodological approaches are suitable for these engagement processes. The Framework Method (Gale et al., 2013; Ritchie and Spencer, 1994; Smith and Firth, 2011) preserves the connection between coded segments and the participant’s full reflective material across days. Longitudinal qualitative research (Saldaña, 2003) extends cross-sectional analysis with within-case change analysis across the intervention’s time arc. A hybrid deductive–inductive coding strategy (Fereday and Muir-Cochrane, 2006) further supports the analytical questions posed. Deductive coding tests theoretical anchors from EFT-C and the close relationships respect literature for empirical fit. Inductive coding provisionally admits emergent material through a watchlist, retaining it on cross-case replication or demoting it on non-replication.

### 1.5 The research aim

The present study reports a qualitative analysis of participant engagement with the EFT-CIDE intervention. We conducted a Framework Analysis (Gale et al., 2013) with a longitudinal qualitative layer (Saldaña, 2003), using hybrid deductive–inductive coding (Fereday and Muir-Cochrane, 2006). Deductive anchors were drawn from EFT-C (Greenberg and Goldman, 2008; Goldman and Greenberg, 2013) and the close relationships respect literature (Frei and Shaver, 2002; Hendrick and Hendrick, 2006; Hendrick et al., 2010; Lawrence-Lightfoot, 2000). The analysis addressed three questions:

*Research question 1.* How well does an a priori framework anchored in EFT-C theory and the close relationships respect literature fit the dataset of EFT-CIDE reflective material?
*Research question 2.* What patterns of within-corpus variability characterise participant engagement, both in longitudinal trajectories through the intervention and in how the same prompt is received by different participants?
*Research question 3.* What do the within-corpus patterns suggest about the boundary conditions for intervention suitability: for whom does EFT-CIDE work as intended, for whom is it suitable as an adjunct, for whom is caution with monitoring warranted, and for whom is it unlikely to produce benefit as a standalone?

## 2 Methods

### 2.1 Design

The study was designed to examine individual participants’ engagement with identity system content, not dyadic interaction. Partner behaviour enters the analysis only as reported by the participant and is treated throughout as the participant’s account rather than as an independent observation of what the partner did (see section 4.5). The study used the Framework Method (Gale et al., 2013) as its primary analytical approach, extended with a longitudinal qualitative layer following Saldaña (2003) to address within-case change across the 14-day intervention window. Coding combined *a priori* deductive anchors drawn from established theory with inductive open codes flagged through a watchlist mechanism, following the hybrid deductive–inductive procedure of Fereday and Muir-Cochrane (2006). The Framework Method (Gale et al., 2013; Ritchie and Spencer, 1994; Smith and Firth, 2011) was preferred over wholly inductive thematic analysis and over interpretative phenomenological analysis because the analytic task was neither open-ended theme generation nor the idiographic reconstruction of a small number of lived experiences, but the systematic comparison of many cases against a partly a priori thematic structure derived from EFT-C and the close relationships literature, with a principled route for admitting emergent material (Fereday and Muir-Cochrane, 2006). The matrix-based, case-by-theme organisation of the Framework Method supports this cross-case comparison while preserving an auditable index that links every coded segment to its source across the 14-day window (Gale et al., 2013; Saldaña, 2003)—a structure that purely inductive thematic analysis does not require and that interpretative phenomenological analysis, given its small-sample idiographic focus, is not designed to provide. Where individual segments are interpreted, they are read as accounts produced within the reflective task rather than as direct measurements of behaviour (see 4.5).

### 2.2 Participants

Sixty participants completed EFT-CIDE and contributed reflective material analysed in this study. Recruitment utilised a convenience panel sample: eligible adults were drawn from the online research panel of an external survey agency between February 2026 and April 2026 in Slovakia. They were approached by email invitation, with opt-in informed consent completed online before enrolment. The parent recruitment procedure and quantitative outcome arm are reported in a separate manuscript (Halamová, 2026). Of 66 initially recruited, 60 completed and contributed indexable material. While the parent quantitative trial applied a minimum engagement criterion (≥12 days of reflection) appropriate for outcome–effect estimation, the present qualitative analysis includes all participants who contributed indexable reflective material (n = 60). This wider inclusion captures the full range of engagement pattern variability necessary to characterise both substantive engagement (Types A–D) and engagement boundaries (Types E–F), which are themselves theoretically informative findings about intervention reach. Eligible panel members who consented to participate completed the baseline assessment online and were then enrolled into the trial via the Self-Growth Institute mobile application^1^, which delivered the daily intervention tasks and hosted all assessment forms. Inclusion criteria required participants to be adults currently in a committed partner relationship of at least 6 months; the only exclusion criterion was being under 18 years of age. A financial incentive of €50 was offered for completion of all three assessment waves, in accordance with the survey agency’s policy. Sample size was guided by the principle of analytic adequacy for qualitative analysis (saturation in code identification was reached during the first three indexing batches, with subsequent batches replicating rather than extending the framework); the final sample of 60 supports the longitudinal trajectory typology with sufficient cell membership in each of the six trajectory types for cross-case interpretation.

Sample sociodemographic and relationship characteristics are summarised in Table 1.

**Table 1:** Sample sociodemographic and relationship characteristics (*N* = 60).

Characteristic	*n*/*M* (*SD*)	% / range; median
Age (years)	38.4 (11.5)	18–74; 36.5
Relationship duration (years)	11.2 (9.7)	1–39; 9.0
Sex		
Women	31	51.7
Men	29	48.3
Relationship status		
Marriage	30	50.0
Unmarried committed partnership	21	35.0
Dating	9	15.0
Cohabitation with current partner	43	71.7
At least one child (any source)	37	61.7
At least one child <18 in household	26	43.3
Education		
Primary or less	4	6.7
Secondary	27	45.0
Post-secondary technical	1	1.7
Bachelor’s	8	13.3
Master’s	18	30.0
Doctoral	2	3.3
Region of residence		
Bratislava	14	23.3
Banská Bystrica	8	13.3
Žilina	8	13.3
Trnava	8	13.3
Košice	8	13.3
Trenčín	7	11.7
Prešov	6	10.0
Nitra	1	1.7

Self-reported nationality: Slovak *n* = 57 (95.0%), Roma *n* = 2 (3.3%), Hungarian *n* = 1 (1.7%). All child counts include biological and adopted children from current and previous relationships. Region percentages are based on *N* = 60.

### 2.3 Intervention

EFT-CIDE (Halamová, 2026) is a 14-day self-guided mobile app intervention developed by the first author within the EFT-C framework (Greenberg and Goldman, 2008; Goldman and Greenberg, 2013) and the close relationships respect literature (Frei and Shaver, 2002; Hendrick and Hendrick, 2006; Hendrick et al., 2010; Lawrence-Lightfoot, 2000), to deliver respect/identity system content.

The intervention organises respect-focused content across fourteen daily tasks. Days 1–3 establish the experiential frame: recalling and experiencing moments of feeling respected (D1), auditing one’s own disrespectful behaviours against a 40-item checklist (D2), and practising empathic validation (D3). Days 4–7 develop appreciation, acceptance, and autonomy: expressing partner-specific appreciation across ten quality domains (D4), receiving compliments and capturing positive partner behaviour (D5), an acceptance process for perpetual differences (D6), and reformulating controlling impulses into autonomy-supportive responses (D7). Days 8–11 build assertive expression of one’s own limits: constructive I-statement complaining (D8), empathic refusal (D9), the “boundary sandwich” distinguishing boundaries from control (D10), and an eight-step collaborative negotiation (D11). Days 12–14 conclude with cycle work and integration: mapping the couple’s negative identity cycle (D12), interrupting shame-driven dominance–submission cycles and replacing them with positive ones (D13), and a values-based identity exercise committing to changes independently of the partner’s behaviour (D14). Each daily task used a four-step reflective prompt structure: EXPERIENCE (immediate affective response to the task), LEARN (key insight or recognition derived from the day’s material), INTEND (committed orientation moving forward), and APPLY (concrete behavioural plan). This four-step sequence was modelled on the experiential–learning cycle (Kolb, 1984), in which concrete experience is followed by reflective observation, abstract conceptualisation, and active experimentation, and on the emotion-focused principle that durable change proceeds from contacting an emotional experience, making sense of it, and translating that meaning into new action rather than from instruction alone (Greenberg and Goldman, 2008; Elliott and Greenberg, 2007). EXPERIENCE anchors the day in the participant’s immediate affective response so that subsequent reflection is grounded in felt experience rather than abstraction; LEARN supports the meaning-making step; and INTEND and APPLY convert that meaning into a committed orientation and a concrete behavioural plan, the action phase through which reflective insight is intended to reach the relationship. After D14, participants could complete a set of POST items reflecting on the cumulative intervention experience and on changes in the relationship over the intervention period.

The intervention was developed in both Slovak and English and is self-administered without therapist contact. Participants completed the daily reflections in writing within the mobile app at a time of their choosing each day. No other intervention contact occurred outside the app.

### 2.4 Data

The dataset comprised text reflections submitted within the mobile app across the 14-day intervention window, plus POST items where completed. Each daily task could produce up to four steps (EXPERIENCE / LEARN / INTEND / APPLY), yielding up to 56 base segments per fully completing participant before POST items; full POST completion added up to seven further segments. Across the 60 participants, the total dataset comprised 2,740 indexed segments. Word counts per participant ranged from 10 to 1,198 (median 392). The analytical record preserves the source material in verbatim Slovak; English translations used in this manuscript are working translations made by the first author for international readability and do not constitute formal back-translation, since the analytical decisions and the per-segment indexing record are anchored in the Slovak source text (see Section 2.6).

### 2.5 Analytic procedure

Data were analysed in stages corresponding to the Framework Method procedure (Gale et al., 2013), with a longitudinal qualitative layer (Saldaña, 2003) integrated at the charting stage. The analysis was conducted by the first author. A documented audit trail was maintained throughout, recording all framework versions, batch memos, decision rules, candidate inductive codes, and methodological flags. Indexing files, codebook versions, and the framework matrix were maintained in Microsoft Excel.

#### 2.5.1 Familiarisation, framework development, and calibration

We first read the full material in batches to familiarise ourselves with the language, register, and recurring content of participants’ reflections. From this familiarisation phase, we developed an initial coding framework anchored in the theoretical sources cited in §2.3. The framework articulated content codes capturing the substantive domains of respect (e.g., active listening, giving and receiving appreciation, boundary setting, cycle recognition, repair); process codes capturing engagement quality signals not reducible to content (e.g., somatic and embodied awareness, vocabulary uptake, partner non-engagement, exit ideation); and a watchlist mechanism for inductive open codes that emerged during familiarisation but had not yet replicated across cases.

Four cases were then selected for calibration coding to test the initial framework against data and produce refined codebook versions. Calibration cases represented a range of engagement levels, content patterns, and apparent trajectory shapes, including one case of sustained partner non-engagement, one of concurrent therapy disclosure, one of templated low engagement, and one of standard content engagement. Each calibration coding pass produced a memo documenting framework adjustments, added decision rules, candidate inductive codes added to the watchlist, and any framework codes flagged for demotion. After the four calibration cases, the framework reached a stable form which was used to begin indexing across the remaining 56 participants.

#### 2.5.2 Indexing

The remaining 56 participants were indexed in six batches, each comprising approximately ten participants. Each batch produced an indexing file that recorded the following for every segment: participant identifier, day, four-step or POST item identifier, exact verbatim Slovak text, primary content code, co-occurring content codes, process code(s), confidence rating (Low / Medium / High), and analytical notes. Each batch was followed by a written memo detailing observed patterns, candidate inductive codes added to the watchlist, framework codes warranting demotion due to non-replication, methodological flags surfaced during indexing, and any cross-batch framework adjustments justified by the cumulative material. Watchlist codes were reviewed after the sixth batch and either integrated into the final codebook or demoted due to cross-batch non-replication; codebook outcomes are reported in §3.2.

#### 2.5.3 Charting (framework matrix)

The indexed material was charted into a participant × theme framework matrix. The matrix positioned each of the 60 participants in cells defined by their predominant engagement pattern, with cell membership derived from the indexed content and process codes rather than externally imposed. Auxiliary sheets recorded code-frequency-per-participant tables, closure patterns, within-participant configurations, and per-participant methodological flags. The framework matrix is available from the corresponding author upon reasonable request (see §2.7).

#### 2.5.4 Longitudinal trajectory layer and cross-case mapping

Within-case longitudinal analysis followed Saldaña's (2003) framework for analysing qualitative change over time. For each participant, we computed day-binned code distributions (early = D1–D5; mid = D6–D10; late = D11–D14), identified inflection points (days marking shifts in voice, affect, or insight), and assigned a trajectory shape. Eight shapes were drawn from established longitudinal qualitative typologies (stable-secure, stable-distressed, deepening, oscillating, front-loaded shift, back-loaded shift, disengaged, fragmented); two further shapes were proposed during analysis to capture configurations that the established typology did not adequately distinguish in this dataset. Trajectory shapes were inductively grouped into higher-order types where similarity in engagement patterns warranted aggregation.

Interpretive synthesis followed the seventh stage of the framework method (Gale et al., 2013), drawing connections within and between categories across cases. The synthesis covered the empirical fit of the framework across cumulative anchor counts and participant-level reach; the three split-pair asymmetries within the boundary, cycle attribution, and repair orientation pairs; bifurcation findings in which the same intervention component produced structurally divergent outcomes across participants; the process code findings; the longitudinal trajectory typology; and the boundary conditions for intervention suitability. The trajectory typology document is provided as Supplementary material; the trajectory analysis spreadsheet is available from the corresponding author upon reasonable request (see §2.7).

### 2.6 Methodological rigour and reflexivity

Several measures supported analytical rigour: documented calibration on four cases before main-sample indexing, with versioned codebook preservation at each revision; segment-level confidence ratings (Low/Medium/High), with low-confidence anchors flagged for review; a watchlist mechanism that required cross-case replication before any inductive code was admitted to the codebook (one graduated, two did not); and working English translations of verbatim Slovak source material rather than formal back-translation, since the analytical record remains anchored in the Slovak source and formal back-translation could introduce drift between analysed material and reported quotations. Item-level rigour reporting is provided in the COREQ checklist (Supplementary material).

Methodological flags surfaced during indexing (within-couple co-completion patterns, concurrent therapy disclosures, religious context for individual APPLY steps, and topic deflection on specific dyadic difficult days) were documented at participant level in the framework matrix (available on reasonable request, see §2.7); specific instances and their interpretive consequences are reported in §3.6 and acknowledged in §4.5. Participant member-checking of findings was not undertaken: in the text data context the analytical record consists of participants’ own verbatim reflective material rather than researcher-generated transcripts, and the trajectory typology analysis was performed at corpus level rather than at individual case level.

#### 2.6.1 Reflexivity

The first author is a senior psychology researcher and certified EFT-C couples therapist; the framework’s anchoring in EFT-C reflects this orientation. The first-person plural (“we”) used throughout reflects the broader research team’s contribution to framework development, methodological consultation, translation review, and manuscript preparation alongside the first author who conducted segment-level coding and trajectory assignment. The first author both developed EFT-CIDE and coded all of the reflective material alone, and this dual role brings clear risks that should be stated openly. Having designed the intervention and worked within the EFT-C model, the first author may have been inclined to interpret the material as showing that the identity system content worked, and to score trajectories as reaching closure when the evidence was actually mixed. Coding alone also meant that no second analyst was there to suggest other readings or to check where a participant’s own words ended and the coder’s expectations began. Several steps were built in to limit these risks, though not to remove them. No inductive code entered the codebook until it recurred across several cases, which prevents categories that appeal to the researcher but are not supported by the data (one code met this bar; two did not and were dropped). Each segment was given a confidence rating, so weakly supported codes were flagged for a second look. Dropping the two weak codes, and keeping the rare minority and non-engagement patterns (Types C, E, and F), shows that material running against expectations was recorded rather than ignored. A full audit trail keeps the record of every decision open to outside scrutiny. These steps help, but only partly: they provide documented transparency, not independent coding, and a second coder remains the proper safeguard for replication (see 4.5).

#### 2.6.2 Reporting standards

Reporting follows the Consolidated Criteria for Reporting Qualitative Research (COREQ; Tong et al., 2007), adapted where appropriate for text data analysis rather than interview data. It is supplemented by the Standards for Reporting Qualitative Research (SRQR; O’Brien et al., 2014) for items not specific to interviews, and the Template for Intervention Description and Replication (TIDieR; Hoffmann et al., 2014) for the intervention description. Completed COREQ and TIDieR checklists are provided as Supplementary material.

### 2.7 Ethics

The study was conducted in accordance with the Declaration of Helsinki and approved by the Ethical Committee of the Faculty of Social and Economic Sciences at Comenius University Bratislava (FSEV 161/2/2023/) on 23 March 2023. All participants provided written informed consent before beginning the intervention. Participation was voluntary and could be discontinued at any time. Data were stored securely and accessed only by the authors and research support staff. Within this manuscript, participant identifiers (P###) are sequence labels assigned during indexing and do not correspond to any external identifying information. Owing to participant confidentiality and the fine-grained nature of the qualitative material (in which combinations of demographic details, relationship context disclosures, and verbatim language could permit identification of individual couples), the full reflective dataset is not publicly deposited. The trajectory typology document is provided as Supplementary material; the framework matrix, trajectory analysis spreadsheet, and verbatim text data are available from the corresponding author upon reasonable request and subject to a confidentiality agreement.

## 3 Results

This section reports findings in six subsections. We first describe the sample’s engagement profile (3.1). We then report the empirical fit of the framework’s content categories with the dataset, supported by anchored exemplars (3.2). Three within-cluster asymmetries that emerged across the boundary, cycle attribution, and repair orientation pairs are reported as a single coordinated finding (3.3). We then report bifurcation patterns, which are intervention components whose outcomes diverged structurally across participants (3.4), followed by process code findings (3.5) and the longitudinal trajectory typology (3.6). Asymmetries describe the shape of typical participant response (one option dominant, alternatives in the minority); bifurcations describe components for which the same intervention task produced two structurally distinct response paths, predictable from prior participant or partner context. All quoted material is presented as working English translations of the Slovak source text; the analytical record preserves the Slovak verbatim, including typographic eccentricities (missing diacritics, typos). Several quotes are provided throughout this section so that each code is tied to real data; a few participants are quoted more than once where their trajectory is discussed in detail.

**Figure 1:**
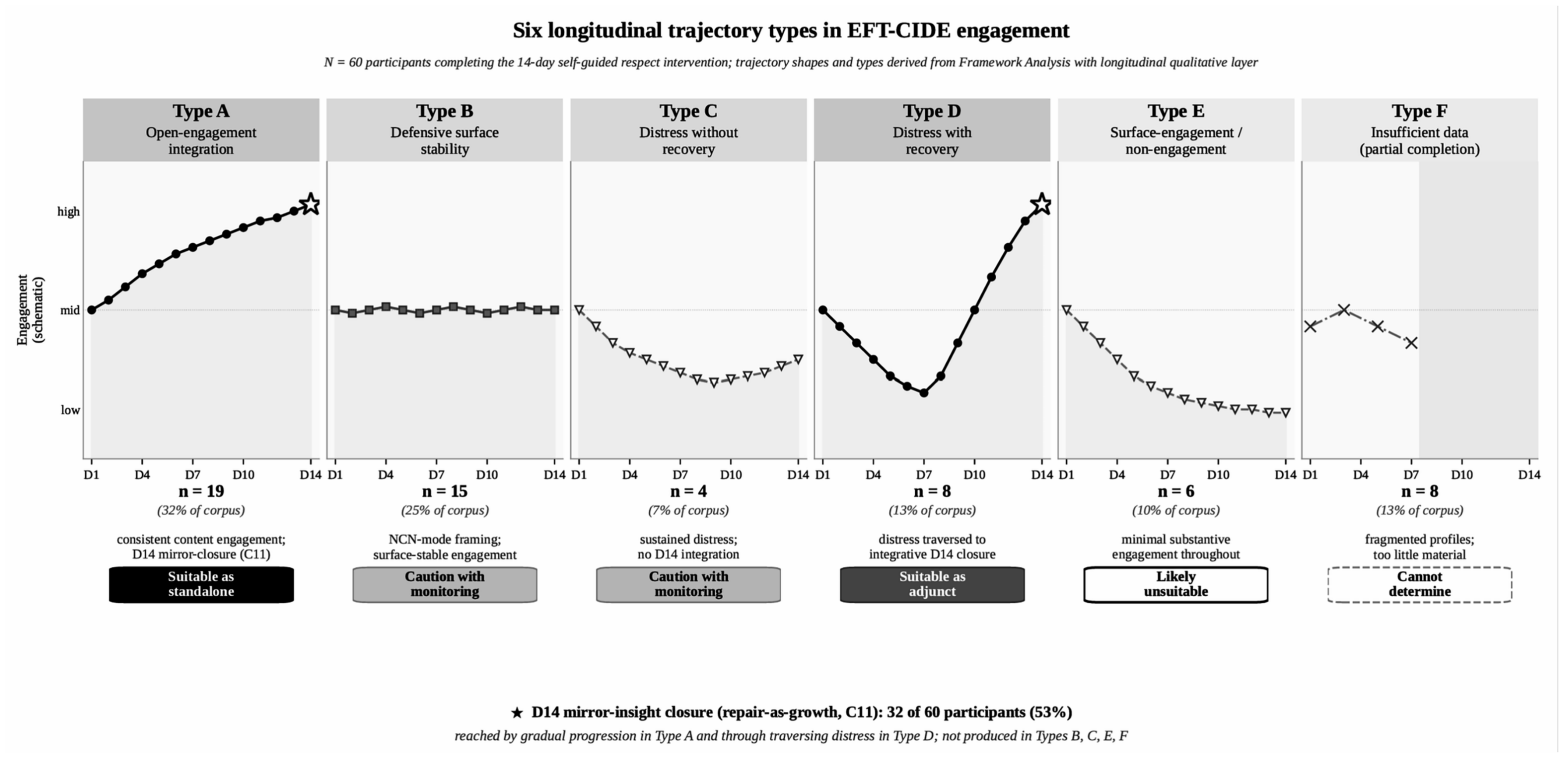
Six longitudinal trajectory types in EFT-CIDE engagement. Six longitudinal trajectory types in EFT-CIDE engagement, derived from Framework Analysis with a longitudinal qualitative layer (*N* = 60). Engagement curves are schematic representations of the predominant within-type pattern across the 14 intervention days; the y-axis indicates relative engagement level (low / mid / high), not a quantified outcome. Each type uses a distinct marker shape and line style (see panels). Star markers at D14 indicate canonical mirror insight closure (C11). Suitability badges below each panel translate the analytical findings into clinical deployment implications across a five-step hierarchy from “Suitable as standalone” (Type A) through “Suitable as adjunct” (Type D), “Caution with monitoring” (Types B and C), “Likely unsuitable” (Type E), and “Cannot determine” (Type F, partial completion). The intervention’s design target, D14 mirror insight closure (C11), was reached by 32 of 60 participants (53%) across Types A and D combined.

Before reporting the findings in detail, we link them to the three research questions from 1.5. The framework fit results in 3.2 answer RQ1: the theory-based framework fits the reflective material well at the level of the main codes, with one new code added from the data and two dropped. The results in 3.3 to 3.6 answer RQ2. The split-pair asymmetries show how the same prompts are usually received; the bifurcations show which parts of the intervention split participants into different outcomes; and the trajectory typology shows how individual participants changed over the 14 days. The trajectory typology and its suitability ratings in 3.6 (with Figure 1) answer RQ3, showing for whom the intervention works as intended, for whom it works as an add-on, for whom it should be used with caution, and for whom it is unlikely to help on its own. Table 2 summarises this, and each of these questions is taken up again in the Discussion (4.1–4.4).

**Table 2:** Mapping of the three research questions to the findings that answer them and the engagement process each bears on.

Research question	Findings that answer it (and engagement process)
RQ1. Fit of the a priori framework	3.2: backbone codes reach 62–90% of participants; one inductive code graduated (C13, trust deficit), two candidate codes appeared in one participant each during calibration, never reappeared in the full dataset, and were therefore removed (C14, C15). Engagement process: which identity system content the prompts make available for uptake.
RQ2. Within-corpus variability	3.3 split-pair asymmetries (how shared prompts are typically received); 3.4 bifurcations (components that fork by participant or partner context as reported); 3.6 longitudinal trajectories (within-case change across 14 days). Engagement process: how the same component is received differently and how engagement moves over time.
RQ3. Boundary conditions for suitability	3.6 and Figure 1: six trajectory types with a five-step suitability gradient (standalone/adjunct/caution with monitoring/likely unsuitable/cannot determine); 32 of 60 participants reached D14 integrative closure. Engagement process: for whom the format produces, supports, or fails to produce benefit.

### 3.1 Sample and engagement profile

Sixty participants contributed indexed material across 2,740 segments. Engagement varied substantially: 17 of 60 participants (28%) completed all 14 days of the intervention, 32 (53%) completed eight to thirteen days, and 11 (18%) completed fewer than eight days. Thirty participants (50%) submitted at least one POST item. Total word counts per participant ranged from 10 (a single-day extreme partial) to 1,198 (a calibration completer with extensive disclosure), with a median of 392 words. Indexed segments per participant ranged from four to 63 (median 52). The content-coded ratio (the proportion of segments receiving a content code rather than only a process code) ranged from below 0.10 in the most templated profiles to above 0.95 in the most engaged, with a sample-wide mean of 0.59.

### 3.2 Content categories: empirical fit and substantive content

The final Codebook retained sixteen content codes after indexing. Two watchlist codes (C14 public-vs-private disrespect asymmetry; C15 developmental and relational schemas) produced no anchors beyond their original calibration occurrences and were demoted due to non-replication. The thirteen retained codes (treating C3a/C3b, C4a/C4b, and C8a/C8b as paired splits) plus the inductively graduated C13 are distributed across the dataset as shown in Table 3.

**Table 3:** Cumulative anchor counts and participant-level reach for the sixteen retained content codes of the codebook.

Code	Operational focus	*n* segments	*n* participants	% of 60
C1	Self-respect awareness	55	23	38%
C2	Active listening and validation	307	54	90%
C3a	Giving appreciation	237	53	88%
C3b	Receiving appreciation	61	30	50%
C4a	Acceptance of partner	95	39	65%
C4b	Autonomy support	68	25	42%
C5	Healthy boundary setting	155	40	67%
C6	Boundary blocked by abandonment fear (internal)	13	10	17%
C7	Boundary met with partner refusal (external)	12	5	8%
C8a	Constructive expression of dissatisfaction	236	51	85%
C8b	Negotiating differences	79	32	53%
C9	Disrespect cycles: mutual contribution recognition	135	37	62%
C10	Disrespect cycles: self-blame loop	15	11	18%
C11	Repair: self-orientation as growth	159	45	75%
C12	Repair: self-orientation as over-responsibility	19	11	18%
C13	Trust deficit/restoration	32	10	17%

#### 3.2.1 Identity system content (C1, C2, C3a, C3b)

The active listening and validation code (C2) was the dataset’s most populous content code, applied to 307 segments across 54 of 60 participants. Substantive content included attentive listening, validation of the partner’s frame even when not agreed with, and refraining from lecturing or correcting. The ‘two-truths’ validation insight surfaced repeatedly as the D3 LEARN-step content; one participant articulated it as follows:

I learned that there is not only one truth: truth is subjective and one must respect and listen to others.

Giving appreciation (C3a; 53 participants) frequently surfaced as a recognition-of-own-deficit framing, with participants identifying under-appreciation as a current state and articulating concrete behavioural commitments to address it. Receiving appreciation (C3b; 30 participants), the counterpart, was notably less prevalent. This giving-versus-receiving asymmetry was consistent with parallel asymmetries observed in the C4a/C4b and C8a/C8b paired splits. P010 D5 LEARN articulated both the cognitive recognition of receiving as practice and the explicit acknowledgement of difficulty:

*That it is important to learn not only to give compliments but also to receive them without minimising… that can be difficult.*

Self-respect awareness (C1; 23 participants) surfaced more often in conjunction with C5 (boundary setting) or C11 (repair as growth) than as a standalone reflective theme.

#### 3.2.2 Difference orientation and boundary content (C4a, C4b, C5, C6, C7)

Acceptance of partner (C4a; 39 participants) frequently distinguished acceptance from resignation explicitly, articulating that accepting stable partner traits did not require relinquishing one’s own perspective. Autonomy support (C4b; 25 participants) showed the lower-side asymmetry pattern characteristic of the paired-split codes (see §3.3).

Within the boundary cluster, healthy boundary setting (C5; 40 participants) was the dominant pattern. The multiplicity-of-modes insight surfaced as a recurring D9 framing for C5; one participant captured it concisely:

*I learned that there are many ways to say no.*

The two block codes, C6 (internal block paired with abandonment fear; 10 participants) and C7 (external block paired with partner refusal; 5 participants), were rare and underscore the within-boundary asymmetry reported in §3.3 (segment-level ratio 13:1:1). C7 co-occurred with E2 (partner non-engagement) in 9 of 12 anchors, suggesting an external block is a partner-side rather than participant-side phenomenon. One participant linked the internal block directly to abandonment fear: “*it was quite hard to realise how difficult it is for me to set boundaries…it strongly connects for me with the fear that I will be abandoned if I really say what is important to me*.”

#### 3.2.3 Communication, cycle, and repair content (C8a, C8b, C9, C10, C11, C12)

Constructive expression of dissatisfaction (C8a; 51 participants) was anchored on D8 (expressing dissatisfaction). Substantive content included the use of I-statements, separation of behaviour from character, and the explicit practice of the intervention’s three-step formula. The normalisation insight that frequently surfaced as the D8 LEARN-step content was articulated as follows:

*That it is natural for us to dislike things about our partner in* var*ious areas.*

Negotiating differences (C8b; 32 participants), the negotiation side of the C8 split, showed the same lower-side asymmetry pattern relative to its expression-side counterpart.

Mutual cycle recognition (C9; 37 participants) was anchored on D12 (cycles) and D13 (stopping cycles), capturing awareness of recurrent patterns in which both partners contribute. A cycle mechanism insight characteristic of the code was articulated as follows:

*I learned that minimising can trigger blame.*

Self-blame loop (C10; 11 participants) was rare and concentrated in three sustained-self-critical participants plus one acute cluster participant; the code anchors the cycle attribution asymmetry reported in 3.3 (segment-level ratio 9:1). The self-blame framing surfaced as shame on reviewing one’s own conduct: “*I was a bit ashamed in front of myself, since I reflect on many situations of disrespectful behaviour toward my partner from my side*.”

Repair as growth (C11; 45 participants) was the dominant D14 closure pattern across the dataset. The mirror insight formulation that recurred across the code’s D14 distribution was articulated by one participant whose D14 closure followed an acute D13 distress episode:

*That often my partner’s behaviour is just a mirroring of my feelings and behaviour, which leads to a negative cycle.*

Repair as over-responsibility (C12; 11 participants) was rare, with two empirical sub-variants, defeated-over-responsibility and self-suppression, anchoring the repair orientation asymmetry reported in §3.3 (segment-level ratio 8:1) and the within-task LEARN/APPLY bifurcation reported in §3.4.

#### 3.2.4 Trust deficit (C13)

C13 was the only inductive candidate code that replicated across the six indexing batches, appearing in enough non-calibration participants to be admitted to the final codebook. Two other candidates (C14, C15) did not replicate beyond their initial occurrences and were dropped. Thirty-two segments coded as C13 across 10 of 60 participants. Substantive content included statements about trust or its absence, trust restoration attempts and their reception, and a partner’s non-reception of restoration efforts. The strongest C13 cluster was concentrated in two participants whose trajectories combined a sustained relationship erosion context with active reflective engagement. C13 routinely co-occurred with E2 (partner non-engagement), particularly with the observed sub-type, in cases of relationship erosion.

### 3.3 Three split-pair asymmetries

**Figure 2:**
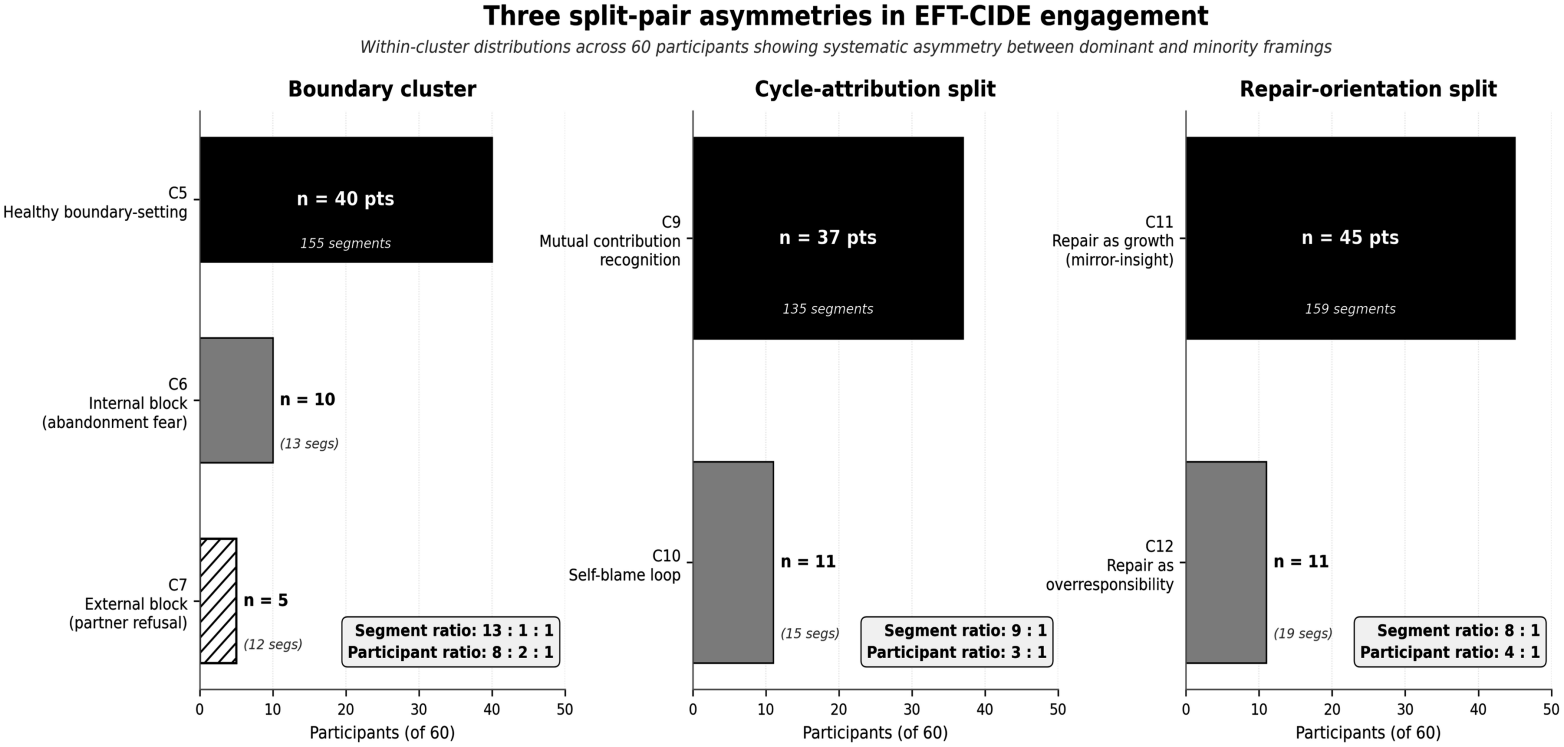
Three split-pair asymmetries in EFT-CIDE engagement. Three split-pair asymmetries within the EFT-CIDE coding framework (*N* = 60). Each panel shows one cluster: boundary setting (left): C5 healthy boundary setting (dominant), C6 internal block paired with abandonment fear, C7 external block paired with partner refusal; cycle attribution (middle): C9 mutual contribution recognition (dominant), C10 self-blame loop; repair orientation (right): C11 repair as growth or mirror insight (dominant), C12 repair as over-responsibility. Bar length indicates the number of participants with at least one anchor for the code; segment counts appear inside dominant bars and beside minority bars. Dominant codes are solid black; minority codes solid medium-grey; C7 is cross-hatched to distinguish the external-block sub-type from C6’s internal-block sub-type within the same cluster. Segment-level and participant-level ratios are inset within each panel. The pattern indicates that most participants produced language consistent with open engagement framings rather than with blocked, self-blaming, or over-responsibility-absorbing framings, and was stable across the six indexing batches.

Three pairs (or triples) of opposing codes in the framework showed a consistent pattern: within each pair, one option strongly dominated and its alternatives appeared only in a small minority of cases. We call these asymmetries because the distribution is structurally lopsided, not 50/50 between the alternatives, but heavily weighted toward one option. The asymmetries occurred in the boundary setting, cycle attribution, and repair orientation clusters of the codebook, and held across all six indexing batches; they represent the dataset’s most empirically robust patterning. See Figure 2 for the three split-pair distributions.

For each asymmetry we report two ratios, which describe the imbalance at different levels:

- Segment-level ratio: across all coded segments in the dataset, what proportion received the dominant code versus its alternatives. This describes how often the dominant pattern was *expressed*.
- Participant-level ratio: how many distinct participants showed the dominant code versus its alternatives. This describes how widely the dominant pattern was *distributed*.

A high segment-level ratio with a lower participant-level ratio means a few participants carried many of the minority anchors; a similar segment- and participant-level ratio means the minority pattern was scattered evenly across cases.

#### 3.3.1 Boundary setting cluster (C5:C6:C7)

Healthy boundary setting (C5) dominated over both block codes (C6 internal block; C7 external block). The segment-level ratio across C5:C6:C7 was approximately 13:1:1, and the participant-level ratio approximately 8: 2: 1. The dominant pattern is, therefore, that participants articulated boundaries clearly and saw the relational impact of doing so; only a small minority encountered either an internal barrier (an inability to refuse despite wanting to) or an external block (a partner’s non-engagement preventing the boundary from landing). C5 anchors were broadly distributed and reached over half the dataset. C6 anchors clustered in participants who directly named fear of relational loss or articulated long-standing personal difficulty with refusal. C7 anchors clustered heavily in a single calibration profile (over half of all C7 segments) with single-anchor surface in four other profiles; C7 co-occurred with E2 (partner non-engagement) in 9 of 12 anchors, suggesting an external block is a partner-side rather than participant-side phenomenon.

#### 3.3.2 Cycle attribution cluster (C9:C10)

Mutual recognition (C9), recognising the negative cycle as something both partners co-produce, dominated over self-blame (C10), attributing the cycle to oneself alone. The segment-level ratio was approximately 9: 1, and the participant-level ratio approximately 3:1. The dominant pattern is, therefore, that participants framed the relational difficulty in mutual rather than self-attributed terms, preserving their own agency while still seeing the dyadic structure of the problem. C9 anchors were broadly distributed across D12 and D13. C10 anchors concentrated in three sustained-self-critical participants who accounted for over half of all C10 segments, plus an acute D6/D7 cluster in one further participant and scattered single anchors elsewhere. The two distributional patterns map onto two distinct configurations of self-recognition: a dominant systemic frame (C9) and a minority self-attribution frame (C10). The minority frame did not foreclose later D14 integration: two of the three sustained-self-critical participants reached C11 mirror insight by the close of the trajectory.

#### 3.3.3 Repair orientation cluster (C11:C12)

Repair as growth (C11), committing to one’s own change with energy and self-respect intact, dominated over repair as over-responsibility (C12), committing to fix the relationship by absorbing more responsibility than is warranted. The segment-level ratio was approximately 8: 1, and the participant-level ratio approximately 4:1. The dominant pattern is, therefore, that participants oriented to repair as growth, not as self-erasure: they took agency for their own contribution without absorbing the partner’s. C11 was the most common D14 closure pattern, applied as a primary code on at least one D14 segment for every participant who reached D14 with content engagement (*n* = 32). C12 anchors were episodic, not sustained: of the eleven participants with C12 anchors, seven received C11 elsewhere in the same trajectory, indicating that C12 framing did not foreclose subsequent C11 integration. The two C12 sub-variants, defeated-over-responsibility and self-suppression, were structurally distinct and warrant independent attention in further analysis.

### 3.4 Bifurcation findings: divergent outcomes from identical intervention components

Five intervention components produced what we term bifurcation patterns: the same prompt elicited two structurally distinct response types, each substantively coherent in itself, with the divide tracking a participant-side or partner-side contextual factor, not random variation. Where the asymmetric splits in §3.3 describe central tendencies (one option dominant, alternatives in the minority), the bifurcations described here describe component-specific *forks*, where the same intervention task functioned in qualitatively different ways for different participants. Each bifurcation is reported with the day, the two poles, the sub-corpus distribution, and a brief interpretive note.

#### 3.4.1 D8, expressing dissatisfaction (I-statement task): skill builder versus stress test

The dominant pole, anchored across most participants, was a *skill builder* outcome: participants produced articulations of the three-step I-statement formula as a usable framework they could apply in their relationship. The minority pole, anchored in three participants with sustained partner non-engagement context, was a *stress test* outcome: the same prompt confirmed that the framework would fail because the partner would not receive it. One participant articulated the LEARN step as *“it gets turned back on me,”* followed by anticipatory anxiety in APPLY. A third sub-pattern, observed in one further participant, was anxious anticipation of harm *to* the partner from the participant’s own use of the framework. The same task led to three structurally distinct outcomes, with the participant’s account of partner engagement associated with which fork a given participant landed on.

#### 3.4.2 D14, integration: mirror insight closure versus no-change-needed continuation

The dominant pole was *mirror insight closure* (C11), an integrative reorientation toward self-change as the leverage point for relational change, observed in 32 of 60 participants (“change starts with me” as growth orientation, not self-blame). The minority pole was *no-change-needed continuation*, surface-stable closure where the no-change-needed framing that dominated the participant’s earlier trajectory (Type B) extended through the D14 LEARN, INTEND, and APPLY steps as *“we have it figured out.”* This was observed in 9 participants. The same closing task therefore produced integrative reorientation in roughly three-quarters of the participants who reached D14 with content engagement, and surface-stable continuation in the rest.

#### 3.4.3 D9, refuse: skill acquisition versus avoidance

The dominant pole was *skill acquisition*, broadly distributed across the dataset. The minority pole was *avoidance*, instantiated in one participant who deflected the dyadic difficult task to non-dyadic content (gym and self-improvement) on both D8 and D9 specifically. Avoidance here functioned as a topic shift, not templated brevity, suggesting active redirection of the prompt away from the dyadic content the task was probing.

#### 3.4.4 D6, acceptance: within-task bifurcation between cognitive uptake and behavioural commitment

Unlike the other bifurcations (which divide *across* participants), one participant’s response bifurcated *within* the same task cycle: the LEARN step articulated acceptance-not-resignation, while the APPLY step committed to suppressing own disagreement. The same participant therefore produced integrative cognitive uptake without behavioural follow-through, a finding that integrative LEARN does not guarantee integrative APPLY, even within a single day’s reflection.

#### 3.4.5 D12, cycles: cycle management reflection versus *in vivo* cycle escalation

This bifurcation was the most clinically significant. Most participants produced *cycle management reflection*, an analytical mapping of the negative pattern from outside the cycle. Two participants produced *in vivo cycle escalation*: the cycle was activated by the task itself rather than reflected upon. One participant reported that the D13 task followed slipping into *“the negative cycle of reproaches itself”;* another reported somatic-panic symptoms during D12. The same task that gave most participants analytical distance gave these participants direct contact with the very pattern the task was asking them to map.

### 3.5 Process codes: engagement quality, partner-side limits, vocabulary uptake

Seven process codes operated transversally to content categories, recording engagement quality and process-level signals not captured by content coding. Cumulative reach was as follows: somatic and embodied awareness (E1), 135 segments / 42 participants (70%); partner non-engagement (E2), 30/8 (13%); engagement quality flag including the no-change-needed sub-typology (E3), 348/41 (68%); vocabulary uptake (E4), 163/41 (68%); exit ideation (E5), 9/5 (8%); affect-only EXPERIENCE response (E6), 382/56 (93%); and engagement fatigue or deferral (E7), 145/36 (60%).

E1 surfaced predominantly as a distress marker but also as positive embodied affect. A representative somatic-distress anchor came from a participant whose D12 cycle task produced panic-level affect, not reflective awareness of cycles:

*During the exercise I felt extremely negative, my heart pounded, my hands trembled, and I had a tendency [to…].*

E2 produced three empirical sub-types: observed non-engagement (partner has refused or rejected), imagined non-engagement (participant predicts refusal without observed instance), and failed-prior-request (participant has previously asked, partner has not changed). The observed sub-type was articulated in a calibration anchor that established the code:

*That my partner will not change; he snaps back that it is in his nature and he cannot be blamed for bad traits or behaviour.*

E3 was the most populous process code. The bulk of E3 anchors (144 of 348) reflected an inductively derived no-change-needed sub-typology with five sub-variants: stability, domain mastery, relational claim (the most populous), already knew, and outward projection. The relational claim sub-variant was articulated as follows:

*With my husband we respect each other and we do not have to do it consciously. It happens naturally.*

E4 captured uptake of intervention-introduced terminology, the most frequent terms being validation, assertiveness, I-statements, and mirroring. E5 captured exit ideation in nine segments across five participants and was theoretically important despite low prevalence. The acute departure pattern was articulated in an INTEND step immediately backtracked in the same-segment APPLY (‘I will not give up yet’):

*Find a new partner.*

E6 (affect-only EXPERIENCE response) appeared in 56 of 60 participants as the most uniformly distributed process code, capturing the systematic surface of brief EXPERIENCE-step affect responses without content engagement. E7 (engagement fatigue or deferral) appeared in 36 participants and surfaced in two empirical sub-variants: cognitive-load fatigue and templated-aphorism deferral. E7 commonly co-occurred with E3 no-change-needed sub-variants in late-week segments.

### 3.6 Longitudinal trajectory typology

The longitudinal layer assigned each of the 60 participants both a trajectory shape and a higher-order trajectory type. Ten trajectory shapes were used: stable-secure (*n* = 12), no-change-needed-defended (*n* = 11; novel shape), fragmented (*n* = 8), oscillating (*n* = 6), disengaged (*n* = 6), back-loaded shift (*n* = 5), stable-distressed (*n* = 4), deepening (*n* = 4), breakdown-recovery (*n* = 3; novel shape), and front-loaded shift (*n* = 1). Two novel shapes were proposed during analysis: no-change-needed-defended captures defensive surface stability dominated by ‘no change needed’ framing across the trajectory, and breakdown-recovery captures a single-day acute distress episode followed by D14 mirror integration.

Shapes were grouped into six trajectory types (see Figure 1). Type A (open engagement integration; 19 participants, 32%) comprised stable-secure and deepening shapes, plus oscillating and back-loaded-shift sub-cases that closed with D14 mirror integration. Participants in Type A produced consistent content engagement with mature D14 mirror closure (C11). Type B (defensive surface stability; 15 participants, 25%) comprised no-change-needed-defended shapes, plus oscillating, front-loaded shift, and back-loaded shift sub-cases; participants in Type B produced surface-stable engagement dominated by no-change-needed-mode framing. Type C (distress without recovery; 4 participants, 7%) comprised stable-distressed shapes; participants in Type C produced sustained distress without D14 integration. Type D (distress with recovery; 8 participants, 13%) comprised breakdown-recovery shapes, plus back-loaded shift, oscillating, and truncated breakdown-recovery sub-cases; participants in Type D traversed acute or sustained distress to integrative D14 mirror or POST recovery. Type E (surface-engagement/non-engagement; 6 participants, 10%) comprised disengaged shapes; participants in Type E produced minimal substantive engagement throughout. Type F (insufficient data; 8 participants, 13%) comprised fragmented profiles with too little material for trajectory inference.

D14 mirror closure (C11) was a cross-cutting marker observed in 32 of 60 participants. Type A participants reached C11 by gradual progression from open content engagement; Type D participants reached it through traversing distress; Types B, C, E, and F typically did not produce C11 D14 material. Within-participant configurations identified in the trajectory analysis layer included sustained-no-change-needed, no-change-needed-with-mid-week-interruption, no-change-needed-with-single-day-breakdown-and-mirror-recovery, engagement-then-no-change-needed late-week shift, pure-no-change-needed-mini with drop-out, sustained-self-critical-with-D14-mirror, sustained-self-critical-with-attachment-fear, acute-self-critical cluster, engaged-while-distressed-with-recovery, engaged-while-distressed-without-recovery, engaged-while-distressed external-context, engaged-while-distressed-with-current-therapy, templated-engagement-with-active-LEARN, and off-task-templating.

To illustrate how the trajectory types unfold across the 14-day window, rather than only as end-states, three contrasting cases are sketched. In a Type A (open engagement) case, P058 engaged steadily from the early days and closed on D14 with an integrative locus-of-control reflection: “that there are still many things I have under control and that depend only on my decision.” In a Type D (distress-with-recovery) case, P062 engaged substantively while in sustained distress through the mid-to-late days, reported acute somatic distress on the D12 cycle task (“my heart was racing”), and reached a self-soothing closure on D14 (“I try to soothe myself from within”)—the distress-to-integration motion that defines the type. In a Type C (distress-without-recovery) case, P024 produced articulate engagement throughout but did not reach D14 integration, closing instead on a sustained trust deficit (POST: “I still cannot trust him”). The three cases show the same intervention producing integration from stability, integration through distress, and engaged distress without within-window integration.

The longitudinal layer reported here operates at the level of trajectory shape and trajectory type assignment across the early (D1–D5), mid (D6–D10), and late (D11–D14) windows. A finer-grained longitudinal treatment—day-by-day code prevalence distributions, formal inflection point identification, and within-participant transition narratives—is beyond the scope of the present analysis and is reserved for a dedicated longitudinal paper.

Methodological flags identified during the indexing close are noted here for transparency. Five suggestive within-couple co-completion cases (P045, P050, P057, P062, P064) were flagged during indexing as potentially completing the reflective material in dyadic co-presence instead of independently; the suggestive cases cluster in or adjacent to Type B and warrant explicit acknowledgement in the limitations. Two participants (P038, P062) made explicit therapy disclosures during the indexing window, contextualising their respective trajectories. One participant (P065) disclosed a religious context (Catholic Eucharistic adoration) for the D4 APPLY step. One participant (P061) produced sustained off-task drift on the D8 (express dissatisfaction) and D9 (refuse) tasks specifically, with non-dyadic content substituted on those days only.

## 4 Discussion

### 4.1 Summary of findings

The aim of this study was to qualitatively examine how participants engage with the content of EFT-CIDE, a 14-day self-guided mobile app intervention targeting the identity system component of couple relationships within the EFT-C framework. While the intervention’s quantitative effectiveness is reported in a parallel manuscript (Halamová, 2026), the in-app reflective text participants produced during the intervention had not previously been examined. We addressed this gap through Framework Analysis with a longitudinal qualitative layer applied to reflective material from 60 Slovak participants (2,740 indexed segments).

Four sets of empirical findings emerged. The *a priori* framework, anchored in EFT-C and the close relationships respect literature, fitted the dataset well at the level of backbone codes: active listening and validation (C2), giving appreciation (C3a), constructive expression of dissatisfaction (C8a), repair as growth (C11), healthy boundary setting (C5), and mutual cycle recognition (C9). Each of these codes reached 62 to 90% of participants, while also revealing low-prevalence minority distributions (C6, C7, C10, C12) and accommodating one inductively derived code (C13, trust deficit) that graduated through the watchlist mechanism.

Additionally, three split-pair asymmetries within the boundary setting, cycle attribution, and repair orientation clusters produced segment-level ratios of approximately 13:1:1, 9:1, and 8:1, respectively. Most participants, therefore, produced language consistent with open engagement framings rather than with blocked, self-blaming, or over-responsibility-absorbing framings – a coordinated three-pair pattern stable across the six indexing batches.

Moreover, several intervention components produced structurally divergent outcomes across participants, rather than a single dominant response. The clearest bifurcation occurred on Day 8 (constructive expression of dissatisfaction): the same prompt registered as a skill builder for most participants but as a stress test for a small minority whose sustained partner non-engagement context confirmed framework failure, not skill acquisition.

Finally, six longitudinal trajectory types organised the dataset, with 32 of 60 participants (53%) reaching the D14 mirror insight closure (C11), the integrative outcome the intervention’s design targets. The intervention did not reach all participants: trajectory types accounting for ~23% of the dataset (Types E and F) showed minimal substantive engagement or insufficient indexable material, and trajectory Type C (~7%) showed sustained distress without within-window recovery.

### 4.2 Theoretical implications: identity system work as a coordinate intervention target

The strong reach of identity system content codes across the dataset (§3.2) indicates that the identity system domain is independently surfaceable through app-mediated reflective intervention. Participants engage substantively with material framed in identity system terms (self-respect, validation, appreciation, boundary setting, growth) without requiring the intervention to first establish attachment system safety as a prerequisite. The findings are thus consistent with frameworks treating identity system processes as coordinate intervention targets rather than as targets accessible only after attachment system stabilisation (Greenberg and Goldman, 2008; Goldman and Greenberg, 2013).

The three split-pair asymmetries (§3.3) together support an empirical reading in which the intervention’s content, when received by most participants, registers as systemic recognition (not self-attack), as growth orientation (not over-responsibility absorption), and as articulable boundary practice (not blocked or refused boundary practice). Within emotion-focused therapy for couples (Greenberg and Goldman, 2008; Wiebe and Johnson, 2016), this distribution fits with the transformation phase functioning as intended when the identity system has sufficient self-soothing capacity to receive the intervention’s mirroring and validation work without collapse. The process closely parallels the perceived partner responsiveness construct in the close relationships literature, where feeling understood, validated, and cared for by the partner functions as the organising relational mechanism (Reis et al., 2004).

Minority distributions also matter theoretically. The eleven participants with C12 (over-responsibility) anchors include cases where self-orientation work registers as absorbed responsibility, not as agency. One D13 reflection illustrates the pattern:

*I will try to find at least one positive thing about him, every day to highlight his effort and not raise my voice. But my partner is non-communicative, I have to “drag” everything out of him, he praises me, if at all, only in ironic spirit, so again the effort and positive approach will only be from my side.*

Goldman and Greenberg (2013) identify this risk pattern as central to identity system intervention misfire; the dataset’s eleven cases empirically substantiate the existence of the risk in self-guided format. The four sustained-distressed (Type C) profiles show that within the 14-day reflective window, one-partner reflective engagement, however substantive, did not produce visible cycle transformation when partner-side dynamics or entrenched trust deficit sustained the cycle. This fits with Greenberg and Goldman's (2008) account that cycle transformation in EFT-C therapy depends on both partners’ participation. Whether sustained one-partner change over longer time horizons can produce reciprocating partner shifts is a question the present 14-day window does not address; the EFT-CIDE intervention reaches one partner within a bounded period, and the trajectory data describe what happens in that period. In EFT-C-related process research, change depends on how clients engage emotionally and relationally with intervention material, so partner readiness and trust context are better treated as moderators of uptake than as fixed prerequisites (Greenberg and Malcolm, 2002; Goldman and Greenberg, 2013; Elliott and Greenberg, 2007).

The findings also extend the close relationships literature on respect in a specific direction. Frei and Shaver's (2002) prototype work, Hendrick and Hendrick's (2006) measurement work, and Hendrick et al. (2010) on respect and the family established the construct’s dimensions; Lawrence-Lightfoot (2000) developed its philosophical articulation. The present analysis demonstrates that the construct’s dimensions are recoverable from app-mediated reflective material over a 14-day window. Respect can be operationalised as a sequence of daily reflective tasks producing analytically tractable text data, not solely as something accessible through extended ethnographic interview or single-occasion self-report. Recovery is selective: backbone dimensions (caring, validating, appreciating, accepting, supporting autonomy) are broadly recovered; minority dimensions (blocked or refused boundary setting; trust deficit; over-responsibility) are narrowly recovered, chiefly in participants for whom the dimension is the dominant lived reality.

The mapping between the intervention’s backbone codes and the respect dimensions catalogued in the close relationships literature is direct, which is expected because the intervention’s daily tasks were built on those dimensions rather than the dimensions being applied only as a post-hoc lens. Active listening and validation (C2) operationalise the attentive listening and partner perspective recognition dimensions (Frei and Shaver, 2002); giving and receiving appreciation (C3a, C3b) operationalise valuing partner qualities and the caring–supportiveness core that Hendrick and Hendrick (2006) place at the centre of the construct; acceptance and autonomy support (C4a, C4b) operationalise accepting differences and recognising partner autonomy and self-determination; healthy boundary setting (C5) operationalises the articulation of one’s own legitimate limits; constructive expression and negotiation (C8a, C8b) operationalise voicing dissatisfaction while refraining from contempt; and mutual cycle recognition and repair as growth (C9, C11) operationalise the view of respect as a cultivated relational practice (Lawrence-Lightfoot, 2000; Hendrick et al., 2010). Because the design was anchored in these dimensions, the analysis uses respect both as the construct the tasks were intended to elicit and as the interpretive lens for reading what participants produced; the broad recovery of the backbone dimensions is therefore evidence that the tasks elicited the content they were designed to elicit, not that an unrelated corpus happened to fit the framework.

### 4.3 Implications for intervention design: bifurcation findings

The bifurcation findings (§3.4) are the dataset’s strongest contribution to intervention design discussion: the same intervention component led to two structurally distinct response paths, with the divergence systematically associated with participant-side conditions, not with intervention-side variation (Lebow and Snyder, 2022). In EFT-C-related process literature, change also depends on how emotionally and relationally meaningful material is taken up, so the same intervention content can function differently depending on the participant’s relational context and level of engagement (Goldman and Greenberg, 2013; Greenberg and Malcolm, 2002). Partner engagement and trust context appear better understood as moderators of response than as absolute preconditions for benefit in self-guided couple interventions, because technology-enabled couple work shows variable uptake and design sensitivity across users (Doss et al., 2017; Roddy et al., 2020; Linardon and Fuller-Tyszkiewicz, 2020).

On Day 8, the central skill builder versus stress test bifurcation is associated with participants’ pre-existing attachment system safety and trust deficit context. Participants without a sustained C13 (trust deficit) or E2 (partner non-engagement) context received the three-step formula for constructive expression of dissatisfaction as a usable framework; participants with that context received the same prompt as confirmation that the framework would fail in their relationship. The two readings of the same intervention component are not different uptake levels of the same lesson; they are different lessons. Intervention design should anticipate that the dyadic difficult component days (D8 expressing dissatisfaction; D9 declining; D11 negotiating differences; D12 managing cycles) will function as stress tests, not skill builders, for users in a sustained partner non-engagement context. For those users, the intervention should provide alternative scaffolding: psychoeducational framing of partner-side limits and explicit signposting towards dyadic or therapeutic resources (Eldridge et al., 2007).

The Day 12 cycle management versus *in vivo* cycle escalation bifurcation (§3.4) is the most clinically significant of the five. The intervention’s design assumes the cycle work prompt is a reflective, not enacting, prompt; the dataset’s two cases establish that this design assumption holds for most participants but fails for a small number, and that the failure is not benign. Intervention design should consider in-app monitoring on the cycle work days for users showing high-distress signals, with optional pause-and-redirect to lower-intensity material (Torous et al., 2021).

The Day 14 mirror insight versus no-change-needed continuation bifurcation (§3.4) reflects different operating modes of the identity system, not different uptake levels. The bifurcation does not on its own indicate intervention failure for the no-change-needed continuation cases; surface stability may be appropriate when the relationship genuinely is functioning, and the material does not allow per-case resolution of this question. The pairing of no-change-needed continuation with templated form (overlap with Type E) does indicate intervention non-penetration in the sub-set where surface stability and minimum effort engagement co-occur.

Taken together, the bifurcations suggest that intervention components vary in their robustness to participant-side conditions. Days 1, 3, 4, 6, and 7 produced relatively uniform skill acquisition outcomes; Days 8, 9, 12, and 14 produced more variable outcomes contingent on pre-existing conditions. The variability is not a flaw of these intervention components but a property of the content they target; dyadic difficult material is by definition more sensitive to dyadic conditions than dyadic positive material. Intervention design should accept this sensitivity rather than seek to eliminate it, and should provide users with the resources to interpret a stress test outcome as informative, not as failure.

### 4.4 Implications for clinical practice: boundary conditions and screening

The longitudinal trajectory typology supports a five-gradation reading of intervention suitability, summarised in Figure 1. The intervention is deployable as a standalone resource for users in the open engagement (Type A) and distress-with-recovery (without current therapy) configurations: identity system engagement is open from the start or traverses distress to integrative D14 closure. For users in the distress-with-recovery (paired with current therapy) configuration, the intervention’s role is adjunct, not primary; this suggests participants with active distress should be screened for concurrent therapy, not away from the intervention itself.

Caution with monitoring is warranted for the defensive surface stability (Type B) and distress-without-recovery (Type C) configurations. For Type B with templated-form overlap, the concern is that the intervention’s form is being completed without substantive engagement. The ‘no-change-needed-defended’ content may mask either appropriate stability or defensive shielding, a distinction the material does not allow per-case resolution. The five suggestive within-couple co-completion cases cluster in or adjacent to Type B and warrant explicit pre-intervention guidance towards independent, not dyadic, completion of reflective material. For Type C, the concern is the inverse: substantive engagement is sustained but the intervention’s self-orientation work cannot transform the dyadic system unilaterally. The intervention should not be deployed as standalone treatment for users whose presenting concern is sustained relational erosion. For these users, partner-side participation, dyadic therapeutic work, or both, are required for the intervention’s content to produce transformation rather than sustained engaged distress.

The surface-engagement/non-engagement configuration (Type E) is unlikely to produce benefit as a standalone. One specific sub-pattern—task-specific off-task drift on the dissatisfaction-expression and refusal days—suggests active topic deflection, not generalised low engagement. Pre-intervention screening that detects task-deflection patterns could direct these users towards different resources. Type F (insufficient data) represents partial completers for whom the dataset does not support a suitability claim either way. The partial-completion rate is itself a potentially informative engagement indicator. If replicated in larger samples, the rate may suggest an upper bound on the population for whom a 14-day daily-reflection format remains tractable.

Across the typology, the dataset suggests that the EFT-CIDE intervention is clearly suitable for approximately one-third of users, suitable as an adjunct to therapeutic scaffolding for a small further fraction, warrants caution with monitoring for approximately one-fifth, and is likely unsuitable as a standalone for a small remainder. These proportions, derived from a self-selected sample of 60, should not be read as estimates of population-level suitability rates; they describe within-corpus distributions and require independent replication. The implication for clinical practice is conditional, not universal. Practitioners considering the EFT-CIDE intervention as a recommendation should screen for three patterns: sustained partner non-engagement context, sustained distress without external scaffolding, and a templated minimum effort engagement style. They should also pair the intervention with explicit framing about the conditions under which it is and is not likely to produce benefit.

### 4.5 Strengths and limitations

Several methodological features support the analytical claims. The dataset is large for qualitative analysis (60 participants, 2,740 indexed segments). Coding combined two approaches: deductive coding anchored in theory, and inductive coding through a watchlist mechanism. New codes were admitted provisionally, then either kept if they replicated across cases or dropped if they did not. The mechanism worked as intended: one inductive code was kept and two watchlist candidates were dropped. This shows that the framework fits the data rather than being imposed on it. Longitudinal analysis (Saldaña, 2003) was integrated with cross-case framework matrix charting (Gale et al., 2013); thus, trajectory shape and type were assigned alongside content coding. Detailed audit trails (versioned codebook, batch memos, confidence ratings for each segment, methodological flags for each participant) make the analysis reproducible even without public data sharing.

Several limitations bound the claims. The dataset is single-language (Slovak) and from one cultural context (sample characteristics are described in the parent quantitative paper). The trajectory proportions and framework fit may not hold in other settings, especially where respect or self-guided reflection operate differently. Relatedly, EFT-C is itself a culturally situated therapeutic model, and the respect, autonomy, and boundary setting content the intervention foregrounds carries assumptions about relational communication and self-disclosure that may be expressed differently under Slovak relational norms than under the largely Western, English-language samples in which the model was developed. The present analysis does not problematise those norms directly; how Slovak conventions around respect, directness, and digital self-disclosure shaped what participants were willing to write is an open question that cross-cultural replication would need to address. The analysis uses only participant reports. Partner behaviour, dyadic context, and interaction patterns enter only through what each participant wrote; thus, the dataset cannot directly describe what partners did. The 14-day window plus POST items shows within-window engagement but may miss slower changes. One participant’s POST report of partner change after the window suggests the within-window picture is incomplete. Coupling these data with longer follow-up would help establish the post-intervention trajectory more fully.

Five participants produced material suggesting within-couple co-completion, clustered in Type B; the flag is documented but cannot be resolved from the text alone, and trajectory interpretation would change if these reflections were dyadic rather than independent. Working English translations are not formal back-translations. Coding decisions stayed anchored in the Slovak source, but the English presentation may introduce small drift between analysed material and reported quotation. Self-selection into a 14-day daily-reflection intervention is itself a limit. Users unwilling to commit to the format are under-represented; thus, the trajectory distribution describes patterns within this dataset, not the wider population. Finally, one coder (the first author) did all segment coding and trajectory assignment. The watchlist mechanism, confidence ratings, and audit trail were intended to support transparency in place of inter-rater reliability assessment. Future replication studies should add a second coder for formal reliability assessment.

### 4.6 Future directions

Four directions for future work emerge from the present analysis. Replication across linguistic and cultural settings is needed to test how robust the trajectory type distribution is and whether the framework fits data beyond the Slovak dataset (Bernal et al., 2009). The framework matrix and trajectory typology can serve as analytical scaffolding for replication studies in other languages. The watchlist mechanism remains open to admit new inductive codes specific to each setting. A second direction is to combine participant-side reflective data with partner-side measurement, either through dyadic completion of the intervention or through partner outcome measures. This would address the limitation that the present analysis captures only one side of the dyad. The five suggestive co-completion cases in this dataset raise an empirical question rather than closing it: when participants complete reflective work together, does the material differ from material produced independently, and if so, how? Longer follow-up windows are also needed. One participant in this dataset reported partner change after the indexing window ended (POST disclosure) (Doss et al., 2019). Whether this kind of post-intervention recovery is rare or common among users initially classified as Type C is an open question that longer follow-up could answer. Targeted redesign of the bifurcation-prone intervention components, especially Day 8 (constructive expression) and Day 12 (cycles), is the fourth direction.

## 5 Conclusion

The respect framework drawn from EFT-C theory and close relationships research fits the app-based reflective material well, with the main dimensions achieving wide coverage across 60 participants and 2,740 segments. Most participants surfaced identity system content, produced language consistent with growth rather than self-blame, and reached an integrative day-14 closure. The same intervention component was received differently by different users: some component days worked as skill builders for participants in receptive relationships and as stress tests for participants who reported sustained partner non-engagement or low trust. The intervention does not reach everyone. Single-partner reflective work cannot transform partner-side dynamics within 14 days, some users receive certain components as stress rather than skill, and a small group does not engage with the format. The findings position self-guided app-based reflective work as one part of a wider intervention toolkit, not a standalone treatment, and offer a foundation for replication, partner-side measurement, longer follow-up, and component redesign.

## Data Availability

The qualitative datasets generated and analysed for this study are not publicly deposited because the fine-grained nature of the reflective material (in which combinations of demographic details, relationship-context disclosures, and verbatim language could permit identification of individual couples) poses a confidentiality risk. The trajectory typology document is provided as Supplementary material; the framework matrix, trajectory analysis spreadsheet, and verbatim text data are available from the corresponding author upon reasonable request and subject to a confidentiality agreement.

## Footnotes

1
www.self-growth-institute.com
