# Supplementary material for: How participants engage with emotion-focused training for couple identity (EFT-CIDE), a 14-day self-guided mobile app intervention: a framework analysis
Source: Front Psychol. 2026 Jul 15;17:1877423. doi: 10.3389/fpsyg.2026.1877423 (PMC13416257; doi:10.3389/fpsyg.2026.1877423)
Supplement: Supplementary file 1 [file Supplementary_file_1.docx]

**EFT-CIDE Trajectory Typology**

*Phase 5b longitudinal layer per Saldaña (2003) — six trajectory types across 60 participants*

**Overview**

This typology clusters the ten trajectory shapes assigned in EFT-CIDE_Trajectory_Analysis.xlsx into six trajectory types based on underlying engagement pattern with the EFT-CIDE intervention. Trajectory shapes are surface descriptions of within-case change; trajectory types are interpretive groupings that link surface patterns to the underlying motivational-system engagement that EFT-C theorising would predict.

The typology is constructed on the corpus of 60 indexed participants (4 calibration + 56 Phase 4b indexed; ~2,740 segments coded under v3). Each type below specifies defining features, 3-5 canonical example participants, and a theoretical interpretation linked to the Greenberg-Goldman (2008) three-motivational-systems framework: identity, attachment, and attraction.

**Type A — Open-engagement integration**

**Defining features.** Identity-system engagement remains open across the trajectory; engagement form is stable or progressively deepening; mature D14 mirror-closure (C11) typically present; no major distress signals; no nothing to change need dominance. The participant takes in intervention content, processes it reflectively, and integrates the master message ('change starts with me') as growth orientation rather than overresponsibility.

**Trajectory shapes included.** stable-secure (12) + deepening (4) + open-oscillating sub-set (P049, P058 — alternation around content rather than around distress). ~19 participants (32 % of corpus), the most populous type.

**Canonical examples.** P028 (calibration; clean stable-secure with D14 C11 mirror); P051 (deepening with cleanest D14 mirror in corpus, 'I cannot change my partner, only myself'); P038 (calibration; deepening trajectory through concurrent therapy disclosure); P055 (deepening with attachment-fear context layered on identity-system self-recognition); P060 (stable-secure with explicit male emotion-expression learning D4).

**Theoretical interpretation.** This type represents the EFT-C transformation phase functioning as designed (Greenberg & Goldman, 2008; Goldman & Greenberg, 2013). The identity system has sufficient self-soothing capacity to receive the intervention's mirroring and validation work without collapsing into self-attack or shutdown. The C11 mirror-insight at D14 ('I cannot change my partner, only myself') corresponds to the canonical mature transformation outcome in identity-system EFT-C work — a self-orientation that preserves rather than absorbs partner agency. P055's sub-pattern is theoretically distinctive: identity-system self-recognition layered on explicit attachment-system anxiety ('I am afraid to stay alone') yet still producing integrative behavioural change in POST. This dual-system engagement — identity work proceeding while attachment-fear is acknowledged rather than denied — is the EFT-C model's strongest indicated pattern for clinical work.

**Type B — Defensive surface stability**

**Defining features.** Engagement form is surface-stable but content framing is structurally defensive; the participant claims no-change-needed across selective domains, the relationship as a whole, or both. Minimal disclosure of vulnerability; minimal emergence of new codes across the trajectory. Often paired with templated form (single-phrase substitution across INTEND/APPLY).

**Trajectory shapes included.** nothing to change need-defended (11) + defensive-oscillating sub-set (P048 nothing to change need-with-C13-leakage, P050 templated nothing to change need with D13 distress signal) + front-loaded shift (P056 D1 nothing to change need → D2 self-recognition partial opening). ~15 participants (25 % of corpus), the second-most populous type.

**Canonical examples.** P017 (sustained nothing to change need-stability across 13 days, anchor for defensive-stable type); P039 (articulate male canonical nothing to change need-relational-claim); P034 (multi-sub-variant nothing to change need, stability + already-knew + relational-claim); P045 (sustained nothing to change need with co-completion methodological flag); P031 (strongest mixed nothing to change need profile).

**Theoretical interpretation.** This type is theoretically the most interesting in the corpus and the one EFT-C framing illuminates most distinctively. The Greenberg-Goldman (2008) identity system can function in two modes during couple-intervention work: open-engagement (Type A) or protect-mode (Type B).

Protect-mode functions to shield the relationship from threat: if the identity system asserts that no change is needed, the attachment system is correspondingly not destabilised by the intervention's invitation to surface vulnerable primary emotion. Per Goldman & Greenberg (2013), identity-system shielding can be appropriate (when the relationship genuinely is functioning) or defensive (when shielding forecloses needed work). The corpus does not allow us to reliably distinguish these two readings at the participant level — but the systematic pairing of nothing to change need-defended trajectories with templated form (overlap with Type E) suggests the defensive reading is more empirically common. P056's front-loaded shift (D1 nothing to change need → D2 self-recognition) is a partial-opening within Type B and points to the conditions under which protect-mode can yield to open-engagement: a brief but pointed intervention prompt (D2 task probing own contributions to disrespect) penetrates the surface.

***Methodological caveat.*** *Five suggestive within-couple co-completion cases in the corpus (P045, P050, P057, P062, P064) cluster in or adjacent to Type B. Co-completion as a social-desirability driver of the relational-claim sub-variant cannot be ruled out; the manuscript's limitations section should acknowledge this.*

**Type C — Distress-without-recovery**

**Defining features.** Attachment-system content (trust deficit, abandonment fear) is prominent in the participant's reflective material throughout the trajectory; the identity-system intervention is engaged with substantively but does not produce an integrative D14 mirror within the 14-day window. The pattern characterises four participants (~7%) and bounds the intervention's reach in one-partner self-guided format rather than indicating attachment-primacy in the broader theoretical sense.

**Trajectory shapes included.** stable-distressed (4 participants); selective overlap with stable-distressed-leaning oscillating. ~4-5 participants (7-8 %).

**Canonical examples.** P024 (calibration anchor; sustained 22-year-marriage E2/C13 profile, POST 'I still cannot trust him'); P029 (sustained engaged-while-distressed without D14 mirror integration); P053 (D11 explicit despair 'I have not learned anything' followed by D14 observed-rejection frame; follow-up disclosure on long-term changes in partner showed recovery beyond the indexing window); P005 (sustained-departure-leaning with E5 anchors, no recovery within window).

**Theoretical interpretation.** This type is the canonical illustration of the Greenberg and Goldman (2008) claim that negative interactional cycles require both partners' participation to transform. One-partner engagement with strong reflective capacity (the participants in this type are articulate and produce substantial content) is insufficient when the cycle is sustained by either partner-side dynamics (observed E2) or by entrenched trust-deficit that the intervention's identity-system work cannot reach unilaterally. The absence of D14 mirror integration is theoretically meaningful: the participant has the cognitive material for self-orientation (C11) but the ongoing relational reality contradicts the integrative move. P053's follow-up disclosure on long-term changes in partner ('Slowly my wife's behaviour is changing') is important — it suggests post-intervention recovery is possible even when within-window recovery is absent, perhaps as the participant's sustained behavioural change eventually shifts the partner's stance. The window during which Phase 4b indexes the trajectory may underestimate eventual transformation. This points toward the EFT-C framing of intervention work as initiating processes that complete on longer timescales than the protocol itself.

**Type D — Distress-with-recovery**

**Defining features.** Acute or sustained distress within the trajectory yields to integrative D14 mirror-insight or to POST positive shift. The trajectory traverses distress to insight rather than ending in either state. May involve a discrete breakdown episode (breakdown-recovery shape) or a gradual late-phase reframing (back-loaded shift toward integration).

**Trajectory shapes included.** breakdown-recovery (3) + back-loaded-shift sub-set with distress-to-integration motion (P002, P052, P018) + recovery-oscillating sub-set (P062 canonical case). ~8 participants (~13 %).

**Canonical examples.** P041 (acute D10/D12 departure-ideation backtracked in same-segment APPLY then D14 mature 'it is simplier to start by changing myself'); P063 (nothing to change need-domain-mastery with D13 single-day breakdown 'negative cycle of blaming' followed by D14 the most striking distress-to-mirror integration in the corpus 'partner's behaviour is just mirroring mine'); P062 (strongest engaged-while-distressed-with-current-therapy in corpus, D8-D13 sustained distress including D12 somatic panic 'my heart was racing', D14 self-compassion recovery 'I try to soothe myself from within'); P044 (humour-defence D2 ironic exit-ideation with positive shift on the immediate post-intervention disclosure of changes in self); P052 (D14 failed-prior-request E2 frame integrated with C11 'Change could be only within my reactions').

**Theoretical interpretation.** This type is the canonical illustration of the EFT-C transformation phase as Greenberg and Goldman (2008) and Goldman and Greenberg (2013) describe it for couples work — primary vulnerable emotion (e.g., sadness, fear) being metabolised into integrative self-orientation. The within-participant motion from distress to mirror is the precise pattern EFT-C theorising predicts when the identity system has sufficient reflective capacity to soothe rather than collapse under attachment-system distress. The breakdown-recovery sub-shape (P041, P044, P063) is theoretically the most striking: a discrete acute distress peak can be transformed into integrative insight within a single day's reflective cycle when the identity system holds. P062's case is theoretically distinctive in a different way — the explicit therapy disclosure (D13 'I will again start psychotherapy') indicates that the participant's identity-system reflective capacity has external scaffolding from concurrent therapeutic work. The intervention is functioning here as adjunct to therapy rather than as standalone, and the D14 self-compassion outcome is plausibly co-produced by both. This points toward a manuscript discussion of the EFT-CIDE intervention's appropriate role: standalone for participants in Type A and Type D-without-therapy; adjunct for participants in Type C and Type D-with-therapy.

**Type E — Surface-engagement / non-engagement**

**Defining features.** Minimal substantive engagement throughout. Aphoristic, templated, or off-task responses without content depth. The participant fulfils the protocol form (showing up across days) but neither identity-system nor attachment-system is substantially activated by the intervention. POST may be uniformly enthusiastic or uniformly null without referencing intervention content.

**Trajectory shapes included.** disengaged (6) + templated overlap with nothing to change need-defended boundary cases. ~6 participants (10 %).

**Canonical examples.** P008, P016, P033 (standard templated low-engagement); P006 (14-day completer with single-word/aphoristic responses 'Super' / 'OK' / 'Nice' across all days); P057 (pure nothing to change need-relational-claim 2-day mini-profile then drop-out); P061 (most extreme low-engagement with off-task drift on D8 'scratches on the car' and D9 'Go to gym' — the dissatisfaction-expression and saying-no tasks specifically).

**Theoretical interpretation.** This type represents the corpus's clearest example of intervention-form-without-emotional-engagement. Greenberg and Goldman (2008) consistently emphasise that interventions which bypass primary emotion fail to produce transformation; this type empirically demonstrates the converse. The off-task drift in P061 specifically — D8 (express dissatisfaction) and D9 (say no) prompts deflected to non-dyadic content — is theoretically suggestive: the dyadic-difficult tasks are precisely those most likely to activate primary emotion, and avoidance via topic-shift (rather than templated brevity) suggests an active attachment-system or identity-system protection process distinct from generalised low engagement. P061 may therefore sit at the boundary of Type E and Type B (the off-task drift functioning as a defensive operation rather than a non-engagement). P006's aphoristic-throughout pattern is a different sub-variant — the participant is consistent in form but the form does not carry attachment- or identity-system content. Whether this represents a stable engagement style (a pre-existing communication form unsuited to reflective intervention work), a low-literacy or low-effort completion, or a dispositional attribute that the EFT-CIDE format does not penetrate, is not resolvable with current data.

**Type F — Insufficient data**

**Defining features.** Partial completion (typically <6 days completed, often <20 segments) with insufficient indexed material to support trajectory inference. Not a substantive engagement type but a methodological category.

**Trajectory shapes included.** fragmented (8 participants). ~13 % of corpus.

**Canonical examples.** P013 (2 days, 8 segments); P021 (1 day, 4 segments — the briefest profile); P032 (2 days, 5 segments); P046 (5 days with rich D5 C6 + dyad-vs-child anchor but insufficient days); P065 (4 days with D1 'Validation' vocabulary uptake + D4 'Eucharistic Adoration' religious context).

**Theoretical interpretation.** Type F is interpretively a non-trajectory category: the data does not support claims about how the participant engaged with the intervention across time. Some Type F profiles produce theoretically interesting single-day anchors (P046 D5 dyad-vs-child asymmetry; P065 D4 religious context; P021 D1 explicit C13/E5 anchors) that contribute to corpus-level findings but cannot anchor within-case trajectory claims. The 8 Type F cases plus the 6 Type E cases together represent 14 of 60 participants (23 %) for whom the EFT-CIDE intervention did not produce indexable longitudinal engagement — a methodologically important figure for the manuscript's discussion of intervention reach and engagement quality. Per Saldaña (2003), longitudinal qualitative analysis explicitly retains and reports on cases where the longitudinal claim cannot be made; doing so honours the empirical reality rather than overfitting trajectory shapes to insufficient data.

**Cross-type observations**

The trajectory typology and the Phase 5 framework matrix operate at different categorical levels. The Phase 5 cells (nothing-to-change-need-defensive, templated-engagement, sustained-self-critical, engaged-while-distressed, C13/observed-E2) are content-thematic categories; the Phase 5b types (A–F) are trajectory-pattern categories. The two layers cross-cut: the engaged-while-distressed cell, for example, contains members of Type C (P029, no recovery) and Type D (P041, P044, P052, P062 — recovery). The two layers should be reported as cross-tabulated rather than collapsed into a single classification.

Within Type B, the underlying nothing to change need frame's five sub-variants (Codebook v4) are aggregated at the trajectory level because their engagement-pattern signatures are similar (defensive surface stability) even though their content signatures differ. For manuscript-level claims about defensive engagement style, the typology-level grouping is appropriate; for fine-grained claims about which content domains attract nothing to change need framing, the sub-variant level is more informative.
