# Supplementary material for: How participants engage with emotion-focused training for couple identity (EFT-CIDE), a 14-day self-guided mobile app intervention: a framework analysis
Source: Front Psychol. 2026 Jul 15;17:1877423. doi: 10.3389/fpsyg.2026.1877423 (PMC13416257; doi:10.3389/fpsyg.2026.1877423)
Supplement: Supplementary file 2 [file Supplementary_file_2.docx]

**COREQ checklist for the EFT-CIDE manuscript**

*Consolidated criteria for Reporting Qualitative research (COREQ; Tong, Sainsbury, & Craig, 2007). 32-item checklist mapped against the EFT-CIDE manuscript sections.*

**Note on adaptation: COREQ for text-data analysis**

COREQ was developed for in-depth interviews and focus groups (Tong et al., 2007). The EFT-CIDE study uses text-data analysis of participant-submitted reflective material from a self-guided mobile-app intervention rather than interview-based data collection. Items 17–23 of COREQ address interview mechanics and require adaptation for text-data context. In the table below, these items are addressed as follows:

Items addressed in adapted form (intervention-prompt structure, daily-reflection schedule, batch memos, intervention duration, data saturation): items 17, 18, 20, 21 — adaptation noted in each cell.

Items genuinely not applicable to text-data context: items 19 (audio/visual recording), 23 (transcripts returned). Marked N/A with brief explanation.

| **#** | **Item** | **COREQ question** | **Addressed in manuscript** |
| --- | --- | --- | --- |
| **Domain 1: Research team and reflexivity** | | | |
| **Personal characteristics** | | | |
| **1** | Interviewer / facilitator | Which author/s conducted the interview or focus group? | Methods §2.5 — analysis-team composition. *Single-author study; segment-level coding and trajectory assignment performed by the first author. Methodological consultation and translation review provided by broader research team (see §2.6 Reflexivity).* |
| **2** | Credentials | What were the researcher's credentials? E.g., PhD, MD | Methods §2.6 — reflexivity statement. *First author: PhD; senior researcher.* |
| **3** | Occupation | What was their occupation at the time of the study? | Methods §2.6 — reflexivity statement. *First author: Professor of Psychology and certified EFT-C couples therapist.* |
| **4** | Gender | Was the researcher male or female? | Methods §2.6 — reflexivity statement. *First author: female.* |
| **5** | Experience and training | What experience or training did the researcher have? | Methods §2.6 — reflexivity statement. *First author has extensive experience in research and clinical practice, with prior qualitative-methodology publications.* |
| **Relationship with participants** | | | |
| **6** | Relationship established | Was a relationship established prior to study commencement? | Methods §2.3 — "self-administered without therapist contact… No other intervention contact occurred outside the app." Authors had no contact with participants prior to or during data collection. |
| **7** | Participant knowledge of the interviewer | What did the participants know about the researcher? E.g., personal goals, reasons for doing the research | Methods §2.3 + §2.7 — participants knew they were taking part in a research study and provided informed consent (§2.7); authors had no direct contact with participants (§2.3). |
| **8** | Interviewer characteristics | What characteristics were reported about the interviewer/facilitator? E.g., bias, assumptions, reasons and interests in the research topic | Methods §2.6 — reflexivity statement addresses theoretical orientation (anchoring in EFT-C) and procedural safeguards against interpretive bias. *§2.6 Reflexivity statement explicitly addresses the first author's EFT-C theoretical orientation as the framework's anchoring source.* |
| **Domain 2: Study design** | | | |
| **Theoretical framework** | | | |
| **9** | Methodological orientation and theory | What methodological orientation was stated to underpin the study? E.g., grounded theory, discourse analysis, ethnography, phenomenology, content analysis | Methods §2.1 — Framework Method (Gale et al., 2013) extended with longitudinal qualitative layer (Saldaña, 2003) and hybrid deductive-inductive coding (Fereday & Muir-Cochrane, 2006). Theoretical anchoring in EFT-C and close-relationships respect literature. |
| **Participant selection** | | | |
| **10** | Sampling | How were participants selected? E.g., purposive, convenience, consecutive, snowball | Methods §2.2 — recruitment-channel. *Convenience panel sample (online research panel of an external survey agency); now stated explicitly in §2.2.* |
| **11** | Method of approach | How were participants approached? E.g., face-to-face, telephone, mail, email | Methods §2.2 — recruitment-channel. *Approached by email invitation through the online research panel; opt-in informed consent online before enrolment. Now stated in §2.2.* |
| **12** | Sample size | How many participants were in the study? | Methods §2.2 — sixty participants completed EFT-CIDE and contributed reflective material (of 66 initially recruited via the convenience panel sample). Sample size was guided by the principle of analytic adequacy for qualitative analysis; saturation in code identification was reached during the first three indexing batches (see Item 22), supporting the analytical adequacy of the achieved sample. |
| **13** | Non-participation | How many people refused to participate or dropped out? Reasons? | Results §3.1 — completion-rate distribution: 17 of 60 (28%) completed all 14 days; 32 (53%) completed 8–13 days; 11 (18%) completed fewer than 8 days. Methods §2.2 — recruitment-vs-completion ratio: 'Of 66 initially recruited, 60 completed and contributed indexable material.' |
| **Setting** | | | |
| **14** | Setting of data collection | Where was the data collected? E.g., home, clinic, workplace | Methods §2.3 — "delivered in Slovak and self-administered without therapist contact. Participants completed the daily reflections in writing within the mobile app at a time of their choosing each day." Data collection setting was the participant's chosen location for app use. |
| **15** | Presence of non-participants | Was anyone else present besides the participants and researchers? | Methods §2.6 — five suggestive within-couple co-completion cases flagged at participant level in the supplementary framework matrix and acknowledged in limitations. The methodological-flag paragraph explicitly addresses this item. |
| **16** | Description of sample | What are the important characteristics of the sample? E.g., demographic data, date | Methods §2.2 Table 1 — full sample sociodemographic and relationship characteristics: sex (31 women, 29 men), age (M = 38.4, SD = 11.5; range 18–74), relationship duration (M = 11.2, SD = 9.7; range 1–39), relationship status, education, region of residence, nationality. Results §3.1 — engagement profile distribution and within-corpus sex-inference subset (21 of 60 grammatically inferable from Slovak source material during coding). |
| **Data collection** | | | |
| **17** | Interview guide | Were questions, prompts, guides provided by the authors? Was it pilot tested? | Adapted: Methods §2.3 — the EFT-CIDE intervention's daily-task structure (D1–D14) and four-step reflective prompt structure (EXPERIENCE / LEARN / INTEND / APPLY) function as the intervention's standardised prompt set. Each daily task targets a discrete content domain; full prompt content is described schematically in §2.3. |
| **18** | Repeat interviews | Were repeat interviews carried out? If yes, how many? | Adapted: Methods §2.3 — the longitudinal daily-reflection structure across 14 consecutive days plus optional POST items functions as the equivalent of repeated data-collection occasions. Each participant produced reflective material on each day they engaged. |
| **19** | Audio / visual recording | Did the research use audio or visual recording to collect the data? | Methods §2.3 — "Participants completed the daily reflections in writing within the mobile app." *N/A: no audio or visual recording. Data is participant-submitted text.* |
| **20** | Field notes | Were field notes made during and/or after the interview or focus group? | Adapted: Methods §2.5.3 — "Each batch was followed by a written memo describing patterns observed in the batch, candidate inductive codes added to the watch-list, framework codes meriting demotion on non-replication, methodological flags surfaced during indexing, and any cross-batch framework adjustments warranted by the cumulative material." Batch memos function as the analytical equivalent of field notes. |
| **21** | Duration | What was the duration of the interviews or focus group? | Adapted: Intervention duration is 14 days. Per-participant data-volume metrics reported in Results §3.1 (word count range 10–1,198, median 392; segments 4–63, median 52). |
| **22** | Data saturation | Was data saturation discussed? | Methods §2.2 — "saturation in code identification was reached during the first three indexing batches, with subsequent batches replicating rather than extending the framework." Methods §2.5.3 — watch-list mechanism and graduation/demotion outcomes (one inductive code graduated, two demoted on non-replication) further document the saturation argument. |
| **23** | Transcripts returned | Were transcripts returned to participants for comment and/or correction? | *N/A: text data is participant-submitted directly within the mobile app rather than transcribed from recorded interaction. No transcript-return step is applicable. The participant's own writing is the analysed material.* |
| **Domain 3: Analysis and findings** | | | |
| **Data analysis** | | | |
| **24** | Number of data coders | How many data coders coded the data? | Methods §2.5 — analysis-team composition. *Single coder (the first author). Audit procedure: versioned codebook documentation, segment-level confidence ratings (Low/Medium/High), batch memos, watch-list mechanism with cross-batch replication (one inductive code graduated, two demoted). Acknowledged as Limitation 8 in §4.5.* |
| **25** | Description of the coding tree | Did authors provide a description of the coding tree? | Methods §2.5.3 — Codebook described (16 retained content codes including paired splits; seven process codes; NCN sub-typology with five sub-variants). Results §3.2 Table 1 — full code-prevalence summary. Full Codebook provided as supplementary material. |
| **26** | Derivation of themes | Were themes identified in advance or derived from the data? | Methods §2.1 + §2.5 — hybrid deductive-inductive: deductive anchors drawn from EFT-C theory and respect literature; inductive open codes admitted via watch-list mechanism with cross-case-replication requirement. Outcomes documented in §2.5.3 (one inductive code graduated, two demoted). |
| **27** | Software | What software, if applicable, was used to manage the data? | *Methods §2.5 — 'Indexing files, codebook versions, and the framework matrix were maintained in Microsoft Excel).'* |
| **28** | Participant checking | Did participants provide feedback on the findings? | *Methods §2.6 — 'Participant member-checking of findings was not undertaken: in the text-data context the analytical record consists of participants' own verbatim reflective material rather than researcher-generated transcripts of dyadic exchanges, and the trajectory-typology analysis was performed at corpus level rather than at individual-case level.'* |
| **Reporting** | | | |
| **29** | Quotations presented | Were participant quotations presented to illustrate the themes/findings? Was each quotation identified? E.g., participant number | Results §3.2 + §3.3 + §3.5 — verbatim quotations from participants' reflective material presented in English translation; analytical record preserved in verbatim Slovak source per §2.6. Each quotation identified by participant ID (P###), day (D#), and step (e.g., LEARN, INTEND). Methods §2.6 — note on translation status and rationale for English-only presentation in this manuscript. |
| **30** | Data and findings consistent | Was there consistency between the data presented and the findings? | Results §3.2–§3.6 — each finding is anchored to specific codes; verbatim quotations directly support stated findings; aggregate numerical claims (prevalence percentages, split-pair ratios) verifiable against the framework matrix in supplementary material. |
| **31** | Clarity of major themes | Were major themes clearly presented in the findings? | Results §3.2 — content categories presented in three thematic groupings (identity-system; difference-orientation and boundary; communication, cycle, and repair). §3.3 — three split-pair asymmetries as named major findings. §3.4 — bifurcation findings. §3.6 — six trajectory types. |
| **32** | Clarity of minor themes | Is there a description of diverse cases or discussion of minor themes? | Results §3.2 — low-prevalence minority distributions (C6, C7, C10, C12, C13) reported with prevalence and anchored exemplars. §3.3 — minority-frame configurations (C10 sustained-self-critical profiles; C12 sub-variants) elaborated. §3.6 — minority trajectory types (C, E) described. Discussion §4.2 — theoretical implications of minority distributions discussed. |
