# Supplementary material for: How participants engage with emotion-focused training for couple identity (EFT-CIDE), a 14-day self-guided mobile app intervention: a framework analysis
Source: Front Psychol. 2026 Jul 15;17:1877423. doi: 10.3389/fpsyg.2026.1877423 (PMC13416257; doi:10.3389/fpsyg.2026.1877423)
Supplement: Supplementary file 3 [file Supplementary_file_3.docx]

**TIDieR checklist for the EFT-CIDE manuscript**

Template for Intervention Description and Replication (TIDieR; Hoffmann et al., 2014). 12-item checklist mapped against the EFT-CIDE manuscript sections. To be uploaded as a submission supplement alongside the COREQ checklist.

**Note on adaptation**

TIDieR was developed for in-person and most often clinical interventions. The EFT-CIDE intervention is self-guided, app-mediated, and content-fixed; consequently, items addressing personalisation (item 9), modifications (item 10), and provider expertise (item 5) require adaptation for a self-administered digital context. Adaptations are noted in each affected cell.

| **Item** | **TIDieR domain** | **Description / question** | **Manuscript location and statement** |
| --- | --- | --- | --- |
| 1 | Brief name | The name or phrase that describes the intervention. | Title and §2.3. Intervention: Emotion-Focused Training for Couple Identity (EFT-CIDE), a 14-day self-guided mobile-app intervention developed within the EFT-C framework. |
| 2 | Why: rationale, theory or goal | Describe any rationale, theory, or goal essential to the intervention. | §1.2 and §1.3. Theoretical rationale: respect/identity-system processes are theorised within EFT-C as one of three coordinate motivational systems alongside attachment and attraction (Greenberg & Goldman, 2008; Goldman & Greenberg, 2013). Goal: to deliver respect-focused intervention content in self-guided app form, addressing the identity-system component of couple relationships. |
| 3 | What: materials | Describe any physical or informational materials used. | §2.3. Daily reflective prompts and structured psychoeducational materials delivered via the Self-Growth Institute mobile app ([www.self-growth-institute.com](http://www.self-growth-institute.com)), including a 40-item disrespect self-audit checklist (D2), empathetic validation (D3), a ten-domain appreciation framework (D4), an acceptance process (D6), the I-statement formula (D8), empathic refusal (D9), the boundary sandwich (D10), the eight-step collaborative negotiation (D11), and the cycle-mapping and integration prompts (D12–D14). |
| 4 | What: procedures | Describe each procedure, activity, and process used in the intervention. | §2.3. Each daily task uses a four-step reflective prompt structure: EXPERIENCE (immediate affective response), LEARN (key insight), INTEND (committed orientation), APPLY (concrete behavioural plan). After D14, optional POST items invite cumulative reflection. Specific procedural content is described in §2.3, where the fourteen days are organised into four developmental phases (D1–3, D4–7, D8–11, D12–14). |
| 5 | Who provided | For each category of intervention provider, describe expertise, background, and any specific training given. | §2.3. Self-administered without therapist contact. The intervention was developed by the first author (a senior psychology researcher and certified EFT-C couples therapist; see Reflexivity in §2.6). No human provider delivered the intervention to participants. |
| 6 | How: modes of delivery | Describe the modes of delivery (e.g., face-to-face) of the intervention and whether it was provided individually or in a group. | §2.3. Individual delivery via mobile-app interface; participants completed daily reflections in writing within the app. No face-to-face contact, no synchronous interaction with a researcher or therapist. |
| 7 | Where: locations | Describe the type(s) of location(s) where the intervention occurred. | §2.3 and §2.2. The app was self-administered at participants' chosen location and time. Setting: residential / personal device, across all eight administrative regions of Slovakia (manuscript Table 1). |
| 8 | When and how much | Describe the number of times the intervention was delivered, over what period, including the duration, intensity, or dose. | §2.3 and §2.4. Fourteen consecutive daily tasks; one task per day; participants chose the time of day. Each task produced up to four reflective steps (≈56 base segments per fully completing participant) across the 14-day intervention window. Word-count range across the full corpus: 10–1,198 per participant (median 392). |
| 9 | Tailoring | If the intervention was planned to be personalised, titrated or adapted, describe what, why, when, and how. | §2.3. The intervention is content-fixed: all participants received the same 14-day sequence with identical prompts. Personalisation occurred at the response level (participants generated their own reflective text) rather than at the prompt level. No prompt-level tailoring or adaptive sequencing was implemented. |
| 10 | Modifications | If the intervention was modified during the course of the study, describe the changes (what, why, when, and how). | §2.3. No modifications to intervention content occurred during the study period. The intervention was deployed in the form documented and remained stable across the recruitment window (February–April 2026). |
| 11 | How well: planned (fidelity) | If intervention adherence or fidelity was assessed, describe how and by whom, and any strategies used to maintain or improve fidelity. | §2.3 and §2.4. As a self-guided digital intervention, content fidelity is structurally enforced by the app: all participants received identical prompts in identical sequence with no facilitator variability. Engagement-side adherence was assessed at the participant level (days completed; segments contributed; word counts) and reported in §3.1. |
| 12 | How well: actual (adherence) | If intervention adherence or fidelity was assessed, describe the extent to which the intervention was delivered as planned. | §3.1. Engagement varied substantially: 17 of 60 participants (28%) completed all 14 days; 32 (53%) completed eight to thirteen days; 11 (18%) completed fewer than eight days. Thirty (50%) submitted at least one POST item. Indexed segments per participant ranged from 4 to 63 (median 52). Content-coded ratio (substantive vs templated engagement) ranged from below 0.10 to above 0.95. |

**Coverage summary**

All 12 TIDieR items are addressed in the manuscript or in supplementary materials. The intervention's self-guided digital format means items 5 (provider expertise) and 11–12 (fidelity / adherence) operate differently from face-to-face interventions: content fidelity is structurally enforced by the app rather than by training of human providers, and adherence is assessed through engagement metrics (days completed, segments contributed, content-coded ratio) rather than through observer-rated session fidelity.
